# Cardiac Troponin I-Responsive Nanocomposite Materials for Voltammetric Monitoring of Acute Myocardial Infarction

**DOI:** 10.1021/acsomega.4c03252

**Published:** 2024-07-06

**Authors:** Gauri Kishore Hasabnis, Zeynep Altintas

**Affiliations:** † Institute of Chemistry, Faculty of Natural Sciences and Maths, Technical University of Berlin, Straße des 17. Juni 124, 10623 Berlin, Germany; ‡ Institute of Materials Science, Faculty of Engineering, Kiel University, 24143 Kiel, Germany; § Kiel Nano, Surface and Interface Science (KiNSIS), Kiel University, 24118 Kiel, Germany

## Abstract

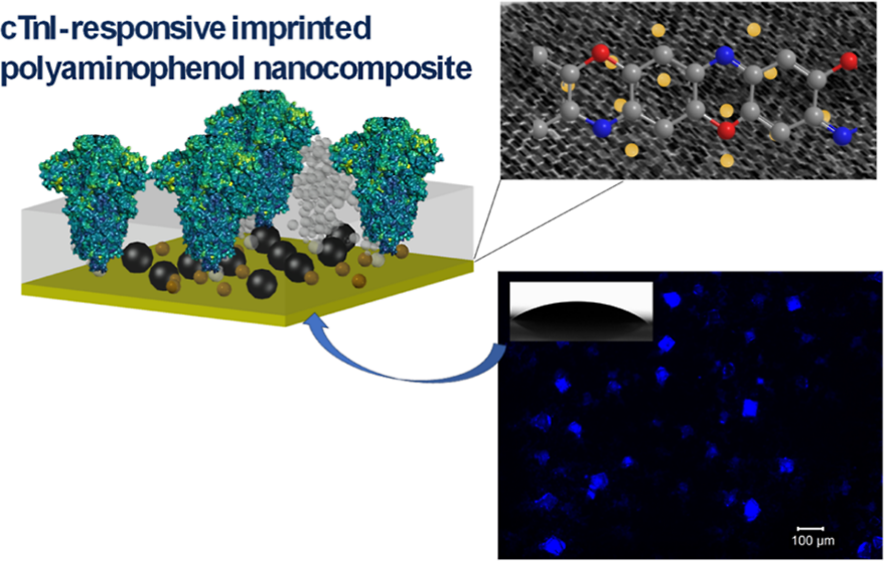

Acute myocardial infarction (AMI) is a severe cardiovascular disease characterized by heart muscle damage due to inadequate blood supply, leading to a life-threatening risk of heart attack. Herein, we report on the design of polyaminophenol-based thin film functional polymers and their thorough optimization by electrochemical, spectroscopic, and microscopic techniques to develop a high-performance point-of-care voltammetric monitoring system. Molecularly imprinted polymer-based cTnI-responsive nanocomposite materials were prepared on an electrode surface by imprinting a specific cTnI epitope, integrating polyaminophenol electrodeposition, along with gold nanoparticles (AuNPs) and graphene quantum dots (GQDs). The characterization techniques, including cyclic and square wave voltammetries, electrochemical impedance spectroscopy, atomic force microscopy, fluorescence microscopy, attenuated total reflection-Fourier transform infrared spectroscopy (ATR-FTIR), and contact angle measurements proved the efficient fabrication of the voltammetric monitoring system relying on cTnI-responsive functional thin films. The sensing platform prepared with the optimized nanocomposite composition of AuNPs, GQDs, and molecularly imprinted polymers exhibited very high sensitivity, reproducibility, specificity, and affinity toward cTnI. The sensor showed a storage stability of 30 days, demonstrating great potential for use in early and point-of-care diagnosis of AMI with its 18 min detection time.

## 1 Introduction

Cardiovascular diseases (CVDs) are the leading cause of global mortality, responsible for 32% of deaths worldwide.^1^ CVDs, such as high blood pressure, coronary artery disease, and rheumatic heart disease, have detrimental effects on the heart and blood vessels.^2^ Acute myocardial infarction (AMI) is a particularly harmful condition, resulting in permanent heart damage and potential fatality. This condition is characterized by restricted blood flow to the heart in the presence of arterial clots, resulting in cardiac insufficiency. During such heart injuries, cardiac biomarkers are released into the bloodstream as a consequence of myocyte necrosis.^3^ Electrocardiography (ECG) is a valuable diagnostic tool for assessing CVDs, including those related to myocyte necrosis. However, comprehensive evaluation of AMI often requires consultation with specialized cardiologists due to the ECG’s limited sensitivity and accuracy.^4^

Elevated levels of specific cardiac biomarkers are crucial for the prognosis of AMI. Cardiac troponin I (cTnI) is considered the gold standard for AMI detection.^5^ In humans, the normal levels are from ≥0.01 to <0.04 ng mL^–1^ of cardiac troponin I (cTnI),^6,7^ whereas if the levels equal to or exceeding 0.04 ng mL^–1^ are considered elevated.^7^ The clinical test outcomes were considered positive when the results surpassed the diagnostic threshold of 0.04 ng mL^–1^ based on cTnI assay.^8^ Detecting cTnI at low concentrations in biological fluids is challenging, but new point-of-care technologies (POCT) are being developed for early AMI diagnosis. The most commonly used technique is enzyme-linked immunosorbent assay (ELISA), which utilizes antibodies as natural receptors.^9^ Despite their delicate structure, limited reusability, and high cost, antibodies are essential in most biological assays for specific protein binding.

To develop more sustainable detection platforms, synthetic receptors like molecularly imprinted polymers (MIPs), aptamers, and peptides have been introduced into various sensors.^10−12^ Among these synthetic recognition elements, MIPs are gaining attention due to their high selectivity, sensitivity, cost-effectiveness, and durability.^13^ MIP synthesis involves developing specific molecular recognition sites within a flexible polymeric network by imprinting a suitable template. After removing the template, the resulting cavities retain the template’s shape and size, exhibiting a similar affinity to natural receptor.^14^ Although the protein imprinting technique is promising, there are few challenges associated with it. In this process, insufficient removal of the template would result in lower selectivity and sensitivity of generated cavities. The affinity of such MIPs could be drastically impaired which could further affect the reliability of the sensor. An epitope (small peptide) is often more structurally stable than a protein. Epitope imprinting provides not only a simple polymerization process with low cost but also overcomes some of the aforementioned difficulties.^15^ Epitope-imprinted sensors enhance sensitivity by attaching epitopes in a specific orientation on a solid support prior to polymerization.^16^

Electropolymerization is a popular method for synthesizing MIPs in sensor preparation.^11,17,18^ It involves the formation of a thin film on the working electrode (WE) surface using an electroactive monomer, and the thickness of the resulting polymeric film can be controlled by adjusting several electrochemical parameters such as the number of cycles, scan rate, and potential range in voltammetry. This method is commonly used in the fabrication of MIP-based electrochemical sensors due to its simplicity and direct control over film thickness.^16^ Furthermore, a large spectrum of nanomaterials such as graphene quantum dots (GQDs), carbon nanotubes, and gold/platinum/silver nanoparticles are being incorporated into nanosensing platforms in order to improve the sensor response. These nanocomposite sensors offer robustness, longevity, and electrocatalytic activity, leading to improved detection limits for specific analytes.^19,20^

Cardiac troponins under normal circumstances are exclusively discharged into the bloodstream from damaged muscle cells during instances of cardiac ischemia. This separation ensures no convergence with skeletal muscle troponins. An array of techniques have been explored to identify troponin proteins, encompassing approaches such as enzyme-linked immunosorbent assay (ELISA),^21^ immunochromatographic assays,^22^ electrochemiluminescence immunoassays,^23^ surface plasmon resonance (SPR),^24^ and quartz crystal microbalance immunosensors.^25^ Nonetheless, these methodologies rely on native antibodies, presenting challenges related to storage stability, sensitivity, and detection thresholds. To surmount these limitations, the incorporation of resilient synthetic analogous in lieu of natural antibodies holds great promise. A particularly encouraging avenue for fabricating artificial receptors is through the application of molecular imprinting technology. As such, MIPs have garnered substantial attention in recent times owing to their cost-effectiveness, heightened affinity, and robust mechanical and chemical stability.

**Scheme 1:**
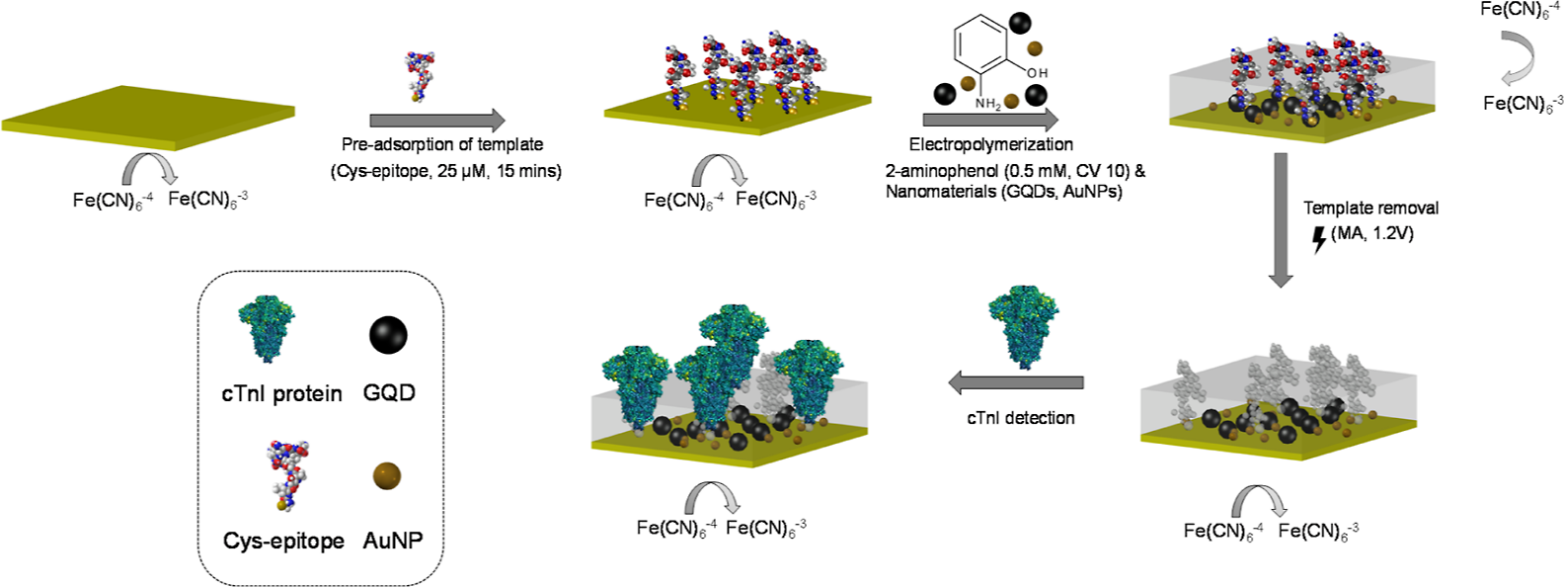
Schematic Representation of a cTnI-Specific Electro-MIP Biosensor Using a Cys-Epitope as a Template, 2-AP, as the Monomer for Electropolymerization, along with GQDs and AuNPs

This study introduces an innovative approach (Scheme 1) to develop pioneering voltammetric MIP sensors. These sensors are built upon thin films of polyaminophenol, with the cTnI-specific epitope serving as the template. The functional monomer, 2-aminophenol, was employed for the precise detection of the cTnI protein. The process involved electrodeposition to create the polyaminophenol film, followed by template removal (TR) to establish complementary cavities within the polymeric structure. These cavities were strategically harnessed to identify both cTnI-derived peptides and the cTnI protein itself, both within buffer solutions and human serum samples. The sensor’s sensitivity and signal amplification were significantly enhanced by the incorporation of key nanomaterials (e.g., GQDs and/or AuNPs) onto the MIP films.

To develop an efficient nanocomposite-based electrochemical sensor, seven different combinations of GQDs and AuNPs were investigated with polyaminophenol thin films. The sensor fabrication steps were characterized using cyclic voltammetry (CV), square wave voltammetry (SWV), and electrochemical impedance spectroscopy (EIS). The chemical functional groups of the optimized MIPs were analyzed using attenuated total reflectance-Fourier transform infrared spectroscopy (ATR-FTIR), while the surface characteristics and topology were studied using atomic force microscopy (AFM), fluorescence microscopy, and contact angle measurements. The best-performing sensor composition has demonstrated great potential to serve the POCT platform with its excellent affinity, selectivity, specificity, cost-efficiency, and storage stability.

## 2 Experimental Section

### 2.1 Chemicals, Reagents, and Electrochemical Techniques

The Supporting Information offers comprehensive details on the reagents and chemicals used, synthesis of nanomaterials as well as the cleaning protocols of electrodes. It also includes the electrochemical measurement parameters (Table S1) employed in this study. In order to determine the optimum parameters for sensor fabrication, a one-factor-at-a-time approach was employed. In this approach, by changing one variable at a time while keeping others constant, the effect of each factor on the outcome can be observed clearly. It is useful when the primary goal is to understand the direct relationship between a single factor and the response variable. Furthermore, in the final stage of the sensor fabrication process, all the optimized parameters were thoroughly examined together.

### 2.2 Optimizing Cysteine-Modified Epitope (Cys-Epitope) on WE

The template molecule plays a critical role in the effective imprinting process, as its shape, size, concentration, and incubation period determine the resulting cavities on the film. In this study, the optimization of the Cys-epitope (CISASRKLQLK) concentration and incubation period was investigated. Two template concentrations (25 and 50 μM) and various incubation periods (15, 30, 70, and 180 min) were examined. The gold wires were incubated with different concentration solutions within these time intervals. Subsequently, the wires were rinsed three times with distilled water to remove weakly bound molecules from the electrode surface. Surface measurements were performed using voltammetry techniques with a redox marker. A multistep amperometry (MA) technique was applied for 30 s to remove the adsorbed template layer. Three different anodic potentials (0.9, 1.2, and 1.4 V) were investigated for voltage optimization, and the surface was recharacterized using CV and SWV to assess the TR process. The obtained electrochemical signals were quantified as relative signal suppression (RSS) using eq S1, as described in the Supporting Information.

### 2.3 Synthesis of Electro-MIP

The cleaned gold wire served as the WE and was connected to the electrochemical workstation. The bare wire surface was characterized using CV and SWV techniques in the presence of a redox solution to establish a reference point for subsequent molecular imprinting steps. Afterward, the wires were incubated with 2.0 mL of Cys-Epi template solution at the optimized concentration of 25 μM and maintained at room temperature for 15 min. Following the incubation period, the wires were carefully rinsed with double-distilled water three times to remove any unbound template molecules from the gold surface. The adsorbed template surface was then characterized using CV and SWV techniques in the presence of a redox marker solution. The MIP synthesis was performed through the electropolymerization of 2-AP, chosen as a suitable functional monomer due to its ease of derivatization and electroactive properties. A 5 mM stock solution of 2-AP was prepared by dissolving it in phosphate-buffered saline (PBS) buffer solution and thoroughly mixing it using a vortex. The final solution was then diluted to a concentration of 0.5 mM of 2-AP. This diluted solution was added to the electrochemical cell and subjected to polymerization using the CV method. Of note, to optimize the polymerization of 2-AP, two electrochemical methods, namely, CV and MA, were considered using various cycles (i.e., CV 20, CV 30, MA 30, and MA 50) to determine the most favorable conditions for polymerization. The efficiency of these processes was evaluated by conducting voltammetry measurements in the presence of a redox marker. The conclusions of the optimization studies are mentioned in Figure S1. Our previous works on epitope imprinting favored the MA method for the electrodeposition of scopoletin-based polymers.^13,26^ Interestingly, the current work highlights the fact that polyaminophenol-based epitope-imprinted thin films require the CV technique for the formation of stable and efficient recognition layers.

### 2.4 Synthesis of Electro-MIP in the Presence of Different Nanomaterial Concentrations

The incorporation of nanomaterials was investigated by examining seven different compositions and concentrations of GQDs and in-house synthesized AuNPs. These nanomaterials were mixed with 2-AP monomer in the prepolymerization mixture which was subsequently subjected to electropolymerization. The synthesis and characterization of AuNPs are explained in previous work.^27^ The process employed for template adsorption, electropolymerization, and their associated parameters remained consistent with those previously detailed in Figure S1. Among the seven evaluated combinations, the inclusion of 250 ppm GQDs, as well as a mixture of GQDs (250 ppm) and AuNPs (7 × 10^13^ NPs mL^–1^), showed promising outcomes. Consequently, these sensors were, respectively, designated as AP-GQD-MIP and AP-GQD-AuNP-MIP. The resulting film underwent thorough characterization by applying a combination of electrochemical techniques, namely, CV, SWV, and EIS.

### 2.5 AFM Characterization of Fabricated MIP-Based Electrochemical Sensors

Surface characterization studies were performed using the AFM Nano Wizard II (JPK Instruments A.G., Germany). The sensor fabrication steps, including the bare gold surface, the self-assembled monolayer (SAM) surface, the templated MIP, the template-free MIP, and the NIP, were evaluated using 2-D height images, cross-section profiles, root-mean-square (rms) roughness, and phase images. Measurements were conducted on a dry gold-coated silicon wafer (Plano GmbH, Wetzlar) at room temperature using the intermittent contact mode. The commercially available AFM probe (TAP300G-G) from Budget Sensors (Innovative Solutions Bulgaria Ltd., Bulgaria) was used with cantilevers having a resonance frequency in the range of 300 ± 100 kHz. The scanning line rate was 0.5 Hz, and the scanning areas of the images were 3 and 1 μm^2^. Image processing was performed using JPKSPM Data processing software.

### 2.6 Contact Angle Goniometry and Fluorescence Microscopy

The contact angles of various stages of the MIP film on SPGE were measured using the OCA 50 Micro goniometer (DataPhysics Instruments GmbH, Germany) equipped with an electronic picoliter dosing system and camera (2048 × 1088-pixel resolution). Fluorescence images were captured using the Keyence BZ-X800 instrument with 10× objectives lenses (Keyence GmbH, Germany).

### 2.7 Fourier Transform Infrared Spectroscopy Analysis

The chemical functionalities of MIPs were analyzed using the attenuated total reflection-Fourier transform infrared spectroscopy (ATR-FTIR) technique. Prior to analysis on the Agilent Cary 630 FTIR spectrometer (GmbH, Germany), the MIP liquid samples were freeze-dried to obtain suitable samples.

### 2.8 Detection of Target Peptide and Protein

In order to establish the sensing method, we initially focused on detecting the original peptide sequence (ISASRKLQLK) of cTnI within a concentration range of 0.01–10 μM. The methodology was then transferred to the detection of the whole protein (cTnI biomarker) across a concentration range of 0.0005–50 ng mL^–1^. Both the peptide and protein samples were meticulously prepared in pH 7.4 PBS buffer. A sequential injection of the samples onto the film surface covered the previously mentioned concentration ranges. Subsequently, the MIP film was incubated for 15 min (for peptide) or 30 min (for protein) before conducting the electrochemical measurements. The SWV technique was employed to measure the target rebinding after incubation, facilitated by the presence of a redox marker. Based on the same detection assay, both sensors (i.e., AP-GQD-MIP and AP-GQD-AuNP-MIP) were used to detect cTnI protein in human serum samples. The cTnI samples were prepared in 50% human serum, mimicking similar concentration ranges, and were subsequently measured using the SWV method. To establish a correlation between the sensor signal and the concentration of the aforementioned samples, the percentage of relative suppression was determined based on the SWV peak points. The signal obtained for TR and the signal obtained from human serum alone were used as reference points for the assays conducted using PBS and human serum, respectively. The equation related to these calculations can be found in the Supporting Information (eq S2). This approach allowed for the quantification of the signal response with the concentration of the target analyte.

### 2.9 Selectivity, Specificity, and Stability

To evaluate the selectivity of the MIP compared to the nonimprinted polymers (NIP), NIPs were synthesized using a similar procedure but without the presence of the template molecule. The affinity of the NIP toward the cTnI protein was subsequently investigated. The rebinding assay of cTnI protein with both NIP and MIP was carried out over a concentration range of 0.05–50 ng mL^–1^. The samples were incubated on the respective polymer surfaces for 30 min, followed by SWV-based measurements in the presence of a redox marker solution. In order to quantify the differences in performance between the MIPs and NIPs, the imprinting factor (IF) was calculated based on these comparative studies. The IF was determined by calculating the ratio of the average RSS percentage between the MIPs and NIPs.

The specificity study was performed for both MIP-based sensors by evaluating the binding profile of the cTnI protein as the target analyte, in comparison to other reference biomolecules including bovine serum albumin, transferrin, p53 protein, and d-glucose, all at a fixed concentration of 100 ng mL^–1^. Each sample was incubated for 30 min and subsequently measured using SWV in the presence of a redox marker solution. The stability test of the best-performing sensor, i.e., AP-GQD-AuNP-MIP, was conducted over a period of 30 days, with a fixed concentration of 10 ng mL^–1^ of cTnI protein. The measurements were performed using CV with a redox marker solution. Data collection was carried out every third day throughout the four-week time frame.

## 3 Results and Discussion

### 3.1 Optimizing Cys-Epitope Adsorption and Removal

The complexity of protein structures poses challenges in the imprinting process, as proteins can undergo drastic conformational changes during polymerization, resulting in less effective cavities for target molecular recognition.^16^ Overcoming these challenges, the cTnI epitope, containing a cysteine group (Figure S2), served as the template for the molecular imprinting process. The inclusion of l-cysteine in the biofunctionalization process is advantageous due to its ability to facilitate well-oriented adsorption on the gold surface (Figure S3). The epitope modification with a cysteine group (–Cys) contributes to the formation of effective cavities in the final molecular imprinting process.^26^ Template concentration is a crucial parameter in the MIP-based biosensor process. Therefore, two concentrations (25 and 50 μM) of the Cys-epitope with varying incubation periods were investigated. Successful preadsorption of the template on the sensor surface was indicated by a high RSS percentage. The highest RSS was achieved after 15 min of incubation with 25 μM Cys-epitope, resulting in a current decrease from 1090 ± 2 μA (bare surface) to 175 ± 0.8 μA (after template adsorption) (eq S1). This condition was correlated to the optimum template adsorption (Figure S4A). The preadsorption of Cys-epitope on the electrode surface was monitored using CV and SWV techniques in the presence of a redox marker solution.

The electrocatalytic TR method was employed to eliminate the peptide in the presence of PBS buffer. A careful investigation of the appropriate potential was conducted to prevent adverse effects on peptide release due to potential cross-linking. Screening experiments for preadsorbed peptide removal are depicted in Figure S4B. It is well-known that amino acids oxidize at a particular potential, and this occurs above 0.7 V for the –Cys group.^28^ The application of electric potential plays a crucial role in cleaving the bond between the template and substrate, resulting in the creation of MIP cavities specific to the target analyte. Upon voltage application, the thiol of –Cys residue may undergo further oxidation,^29^ leading to the formation of disulfide linkage, as illustrated in Figure S5. The electrode surface was characterized using CV and SWV techniques in the presence of a redox marker solution.

Two sets of experiments were conducted, involving template adsorption (25 μM) with incubation periods of 15 and 30 min. In both cases, TR at 0.9 V was found to be inoperable due to the required number of MA cycles, which could potentially damage the electrode surface. Consequently, 1.2. and 1.4 V were selected as the optimal potentials for efficient template detachment and further investigations. Following the application of TR at either 1.2 or 1.4 V, the suppression was completely eliminated (0%), indicating the successful removal of the peptide layer from the gold surface. For subsequent studies, TR at 1.2 V was chosen owing to its efficient TR without compromising the recognition film. The RSS was calculated using eq S1 (Supporting Information), with the bare wire signal serving as the reference point.

### 3.2 Electropolymerization Process Optimization

In the subsequent step, optimizing the thickness of the polymeric recognition film played a vital role in achieving complementary cavities for target recognition. It was important to ensure that the polymer films were both sufficiently thick and flexible, enabling smooth release and capture of the target analyte. The thickness of the film could be controlled by adjusting the number of cycles employed in the electropolymerization technique. The use of 2-AP as an electroactive functional monomer was particularly advantageous in the electropolymerization process. Previous work conducted by Barbero et al.^30^ proposed a mechanism involving the formation of a radical cation [2-AP^+•^] in the initial charge-transfer step, followed by dimerization through either C–C or C–N coupling to generate species (I) and (II), as depicted in Figure S6. The reaction rates and cyclization during electropolymerization determine which species will react faster, favoring the formation of a phenoxazine-like chain structure as the predominant product. However, it is crucial to exercise caution during electropolymerization regarding factors such as concentration, pH, and temperature, in order to avoid potential side reactions, and the formation of complex structured films. In this study, the polymerization process was carried out using a 0.5 mM concentration of the 2-AP solution prepared in PBS buffer. The initial CV cycle revealed a single anodic peak corresponding to the oxidation of the 2-AP monomer, followed by a chain-propagation reaction leading to polymer formation (Figure S7). The absence of a cathodic peak during the reverse scan indicates the irreversibility of the oxidation process. From the second to the tenth cycle, the peak current gradually decreases until reaching saturation. In SWV characterization, the peak current value was significantly decreased compared to the bare signal, indicating the formation of a polymer film with nonconducting nature. Among the options considered in the mapping, CV with 10 cycles was identified as the most suitable method for polyaminophenol (PAP) using preoptimized parameters.

### 3.3 Imprinting Process through Nanomaterials Incorporation

Nanomaterials offer a means to enhance sensitivity in biosensors by amplifying electronic signals and significantly lowering the limit of detection (LOD) by several orders of magnitude.^31^ This heightened sensitivity holds great promise in healthcare and diagnostic applications.^32,33^ In our strategy, GQDs and AuNPs were selected from a range of nanomaterials. AuNPs facilitate improved electron transfer between the biomolecule and the electrode, while GQDs can be functionalized with small nucleic acids and proteins to enhance surface area activity.^34^

In order to optimize nanomaterial composition along with concentration, seven different types of voltammetric MIP-based sensors were examined, and the corresponding optimization results of these sensors are summarized in Figure S8A. It was observed that GQD with low concentration and standalone AuNP (Figure S8A, sensors B, C, and E) could not be optimized due to inconsistent results in TR steps and the lack of reproducibility in experiments. Among the seven combinations of potential sensors tested, the 250 ppm GQDs (AP-GQD-MIP) and the combination of GQDs and AuNP (AP-GQD-AuNP-MIP) were identified as the optimal MIP sensors. These two sensors showed consistent performance and excellent reproducibility in the electrochemical analysis (Figure S8A,B).

**Figure 1:**
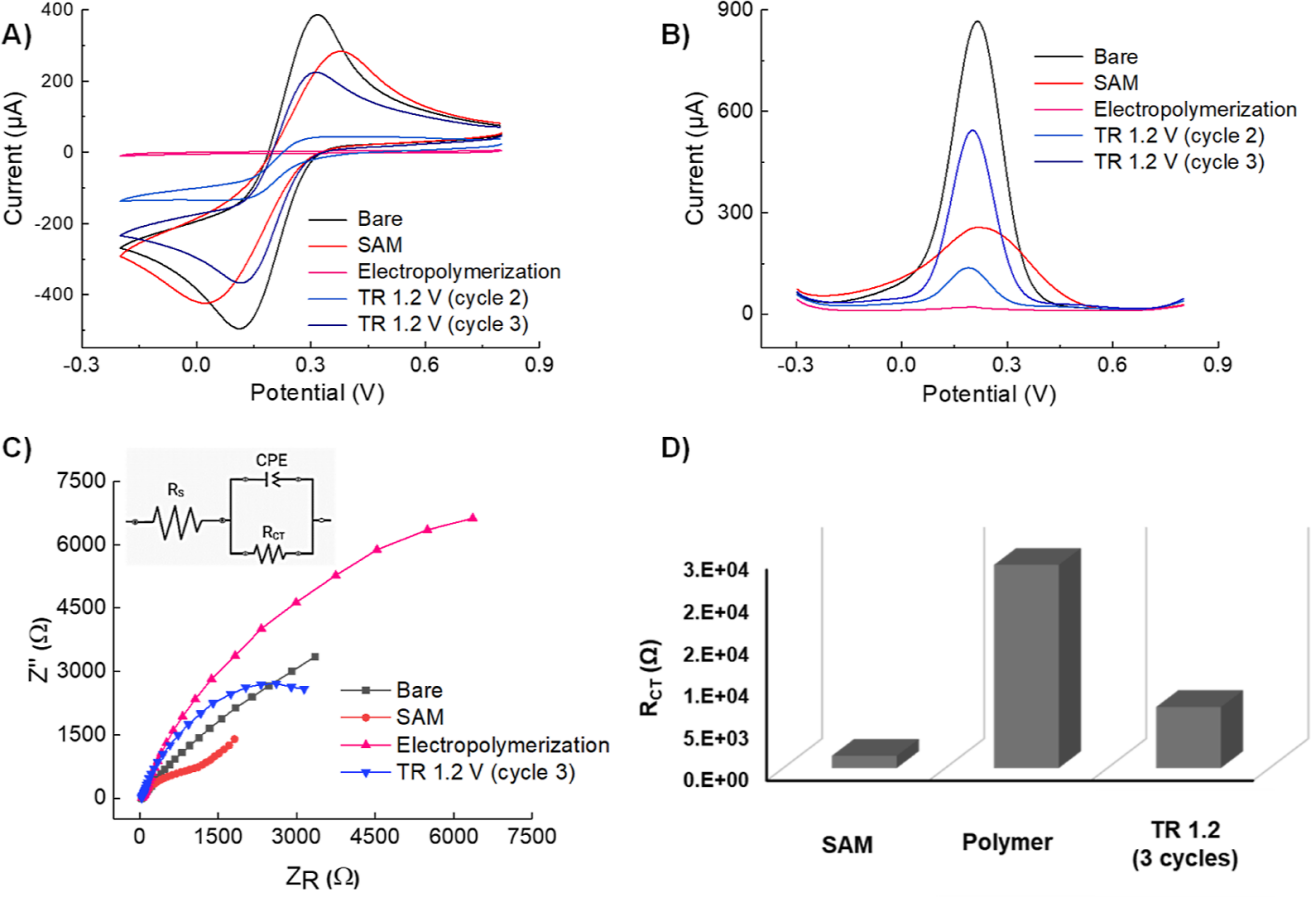
Electrochemical characterization of the AP-GQD-AuNP-MIP sensor fabrication steps was performed using (A) CV, (B) SWV, (C) EIS Nyquist plot, and (D) charge transfer resistance (*R*_ct_) of SAM, polymer, and TR steps (*n* = 3). All electrochemical characterizations were conducted at room temperature using a redox marker solution (10 mM K_3_[Fe(CN)]_6_ with 0.1 M KCl).

The fabrication steps for the sensors are depicted in Scheme 1. The CV and SWV measurements (Figure 1A,B) revealed that the bare signal on the clean Au surface exhibited the highest current peaks (865 ± 3.6 μA) due to the absence of any hindrance. However, the current decreased (240 ± 5.2 μA) when passing through wire owing to the presence of the adsorbed Cys-epitope layer (SAM) compared to the bare signal. Upon the electropolymerization of 2-AP, the current was further suppressed (14 ± 5.5 μA) due to the nonconductive nature of the PAP film that covered the entire surface of the WE.^35^ Nevertheless, the current was restored (546 ± 5.1 μA) after the TR steps, as the redox marker could penetrate the MIP cavities present in the polymeric film. The EIS measurements, specifically the Nyquist plot, provided crucial information regarding the charge transfer resistance and capacitive behavior of the electric double layer.^36^ The EIS data of AP-GQD-AuNP-MIP was analyzed using a simplified Randles circuit (Figure 1C), which contained a charge transfer resistance (*R*_ct_), a constant phase element, and a solution resistance (*R*_s_). Following electropolymerization, the *R*_ct_ value (Figure 1D) increased significantly to 24.1 kΩ, resulting in the characteristic semicircle curve observed in the Nyquist plot. Subsequently, after the TR steps, the *R*_ct_ was dramatically reduced to 7.2 kΩ, indicating an enhanced current passage through the WE. The apparent charge transfer rate constant (*K*_app_) for different electrodes was calculated from their corresponding *R*_ct_ values in the presence of [Fe(CN)6]^3–/4–^ using eq S4 mentioned in Supporting Information.^37^ The value of *K*_app_ is significantly reduced after the formation of the polyaminophenol film. Furthermore, the post-TR increment indicated the possibility of MIP cavities in the film (Table S2). Similar trends were observed for the AP-GQD-MIP sensor, as shown in Figure S9 (Supporting Information).

**Figure 2:**
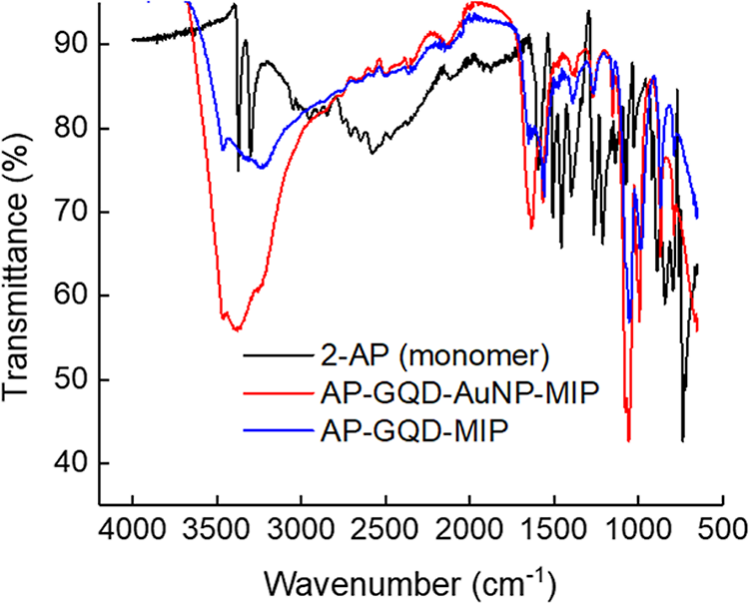
ATR-FTIR spectra of 2-AP (black), AP-GQD-AuNP-MIP (red), and AP-GQD-MIP (blue).

The ATR-FTIR analysis was conducted in order to confirm the polymerization films and gather essential information about the chemical functionality within the MIP films (Figure 2). The monomer 2-AP displayed prominent stretching peaks at 740 cm^–1^, corresponding to a characteristic disubstituted phenyl structure. However, these peaks disappeared after polymerization. The presence of C–N stretch (free amine) at 1265 cm^–1^ and C–O stretch (free hydroxyl) at 1450 cm^–1^ was observed, although their intensities were significantly reduced, indicating the involvement of both functional groups in the formation of PAP. The amino group (−NH_2_) exhibited characteristic bands near 3300 and 3500 cm^–1^, representing the free asymmetrical and symmetrical stretches, respectively. Both polymeric films displayed strong stretching vibrations for functional groups, including C–O–C (1058 cm^–1^, cyclic ether), C=C (1630–1660 cm^–1^), while the peak intensity at 1265 cm^–1^ for C–N stretching was relatively weak. Broad peaks in the range of 3200–3500 cm^–1^ were observed for N–H and O–H stretches, indicating the presence of hydrogen bonding within the polymeric networks. The chemical analysis showed a phenoxazine chain structure (Figure S6), similar to the morpholine moiety formed after the electropolymerization.^38^

### 3.4 AFM Characterization of MIP Films

**Figure 3:**
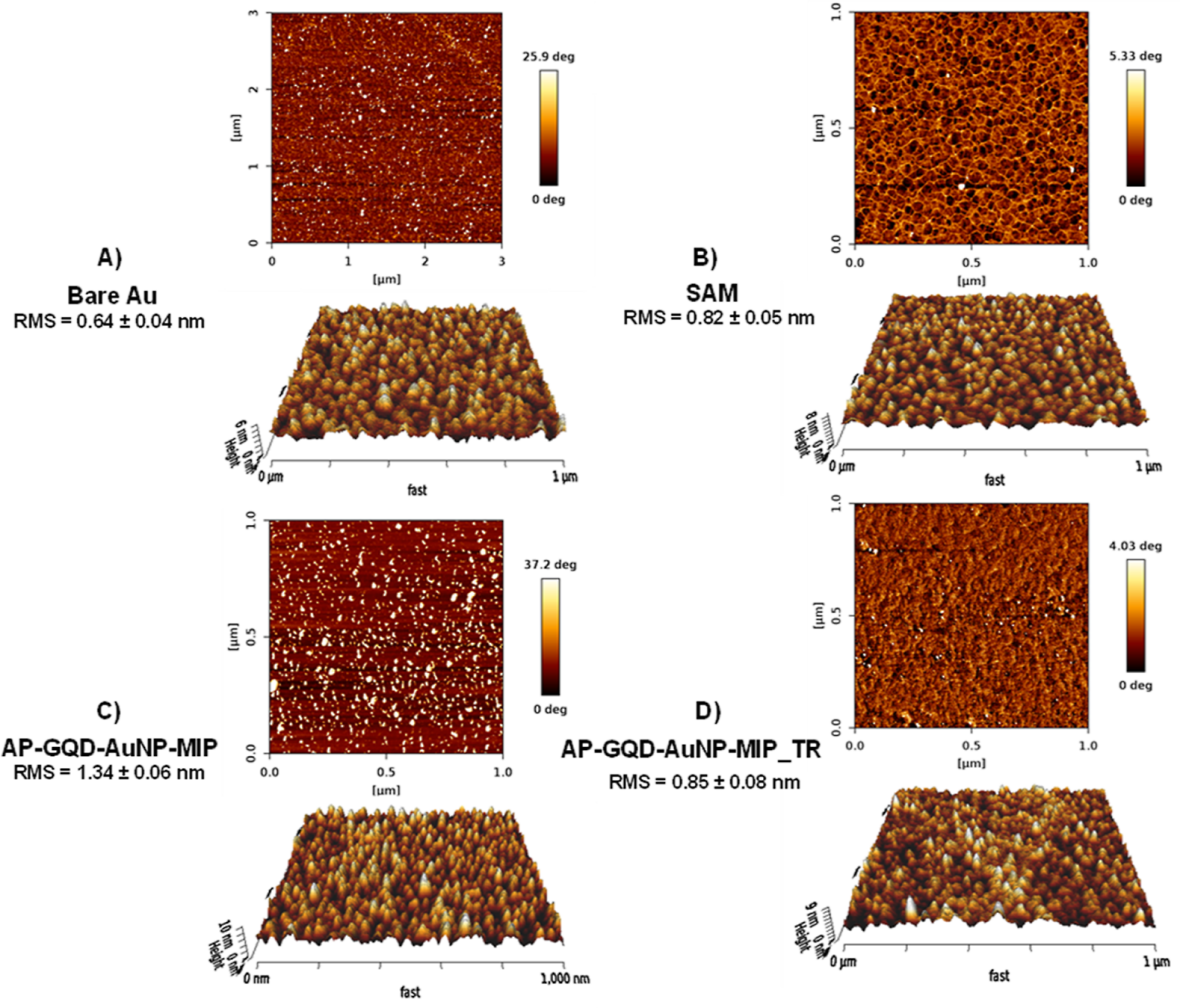
AFM phase images and 3D images of (A) bare Au, (B) template-adsorbed Au (SAM), (C) AP-GQD-AuNP-MIP, and (D) template-free AP-GQD-AuNP-MIP.

AFM was employed to analyze the surface topographies of various samples. Figure 3 presents AFM phase images depicting (A) a bare gold surface, (B) SAM on the gold surface, (C) electropolymerized MIP with the template (AP-GQD-AuNP-MIP), and (D) template-free MIP. The corresponding 3D height traces are also included. The phase images provide a comprehensive understanding of the material properties of thin films.^39,40^ Both sensors exhibited noticeable variations in the poly-AP film (Figure S10). The root-mean-square (rms) roughness of the films significantly increased after electropolymerization due to the presence of the embedded Cys-epitope template. However, upon TR, the film morphology underwent considerable changes, resulting in a reduction of surface rms roughness. In the absence of the template, the NIP film roughness of AP-GQD-NIP remained relatively similar to the SAM surface, although phase analysis revealed visible differences on the surface. The surface roughness of AP-GQD-AuNP-NIP was observed to be smoother compared to the bare surface. However, the AFM phase images and 3D height images clearly showed distinctive compositions on each surface. The removal of template molecules resulted in a smoother surface, attributed to the formation of empty cavities within the polymer network. Additionally, the NIP surface appeared more homogeneous and smoother than the MIP surface due to the absence of the adsorbed peptide. In summary, the AFM results provided valuable insight into the surface topologies and crucial information about the fabrication steps of the sensor.

### 3.5 Fluorescence Microscopic and Contact Angle Analyses of MIP Films

**Figure 4:**
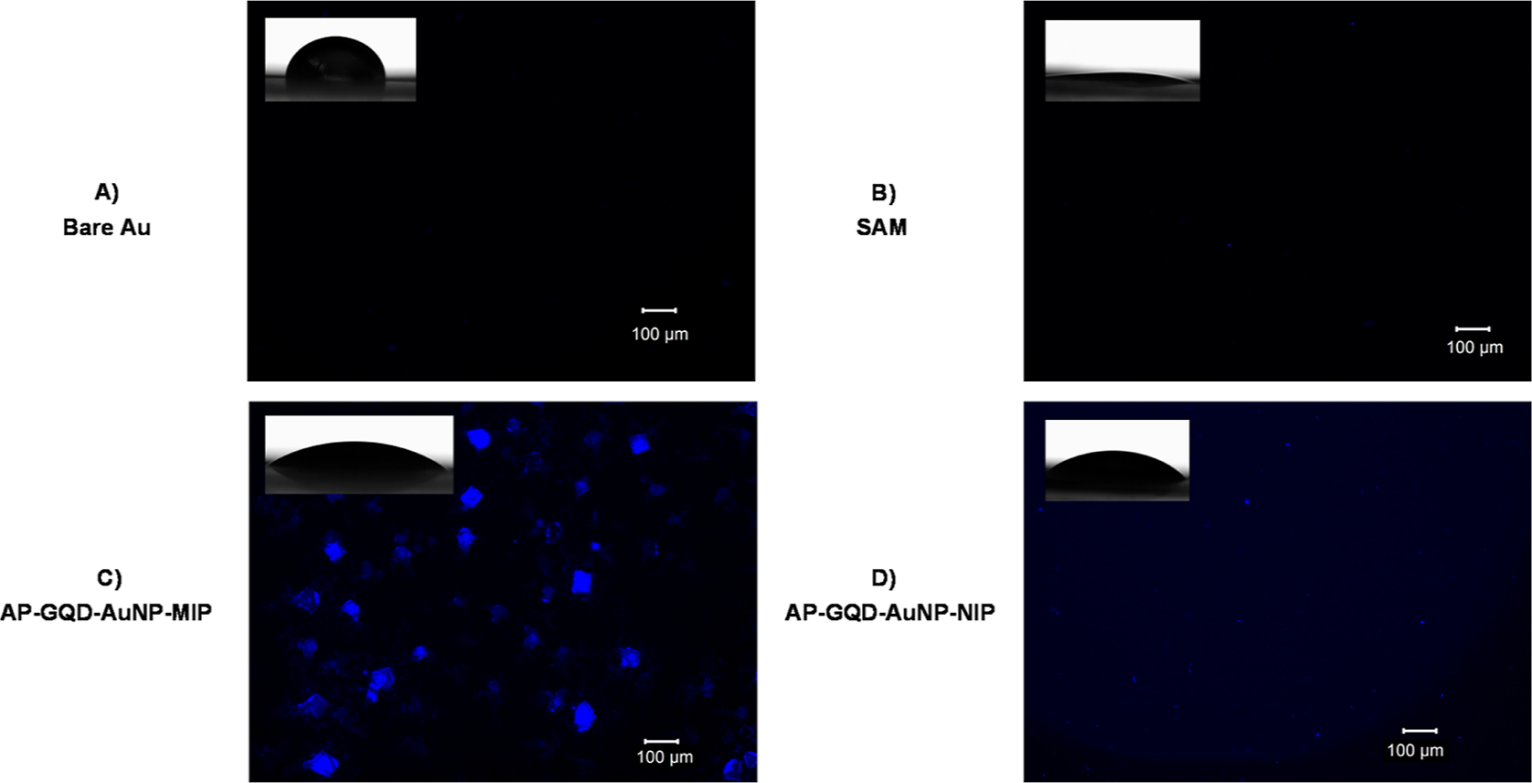
Fluorescence microscopic images with the corresponding contact angle of bare (A), template-adsorbed Au [SAM, (B)], AP-GQD-AuNP-MIP (C), and AP-GQD-AuNP-NIP (D).

The contact angle of the bare gold surface was measured as 88.2°, indicating its hydrophobic nature and lack of fluorescence material (Figure 4A). However, upon adsorption of the Cys-epitope, the gold surface exhibited a significant change in static angle, becoming highly hydrophilic (8.95°), as shown in Figure 4B. This increase in hydrophilicity is likely due to the assembly of cysteine’s –SH groups on the gold surface, forming a uniform layer of Au–S bonds.^41^ Also, the templated surface showed no fluorescence properties since the peptide lacked a fluorophore moiety. When comparing the contact angles of AP-GQD-AuNP-MIP and AP-GQD-AuNP-NIP films, it was observed that the former (30.1°) exhibited higher hydrophilicity than the latter film (40.4°). In the case of NIP films, low fluorescence was detected, and the contact angle indicated a more hydrophobic nature, which can be correlated to the absence of a hydrophilic template. Similar results were observed for AP-GQD-MIP and its corresponding NIP films (Figure S11).

### 3.6 Detection Assays with cTnI Epitope and Protein

The sensing performance was enhanced upon the incorporation of GQDs into the poly(*o*-aminophenol) film, permitting the π–π stacked interactions;^42^ therefore, 2-AP monomer could be fixed on the polymeric film with the aid of GQDs. The incorporation of AuNPs into the sensor led to a further improvement in electron transfer during cTnI detection, attributed to the increased functionality of the polymer. The developed nanocomposite sensor demonstrated excellent analytical performance, providing high sensitivity, selectivity, and durability.

The AP-GQD-MIP and AP-GQD-AuNP-MIP sensors were initially tested for detecting the cTnI epitope (ISASRKLQLK) in PBS buffer to evaluate the recognition capability of electro-MIPs for small peptide and their corresponding cavities. The sensor response was determined as RSS compared to TR using eq S2. After the 15 min incubation, the resulting electrochemical signal was recorded using a redox marker solution.

**Figure 5:**
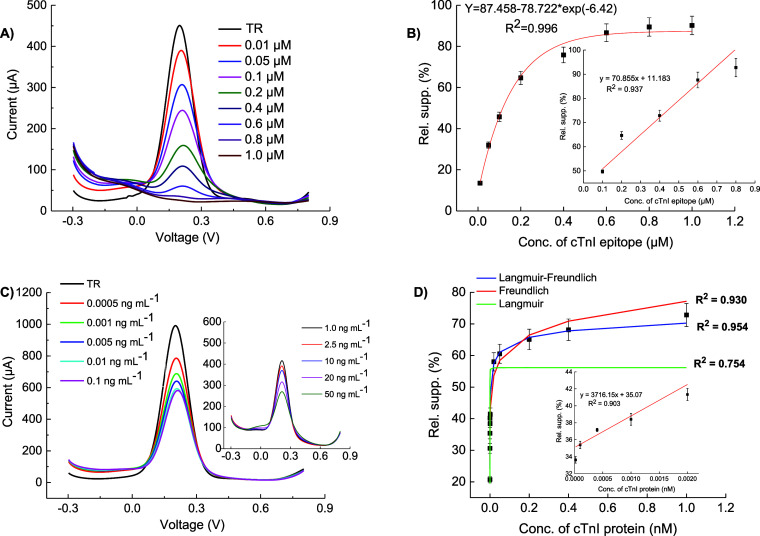
cTnI epitope rebinding assay (A), its concentration dependency as well as linear range (0.1–0.8 μM) (*n* = 6) (B), cTnI protein detection (C), and corresponding binding isotherm with linear regression fit (inset) (D) of AP-GQD-AuNP-MIP (*n* = 6). The Langmuir–Freundlich model (blue curve) was the best fit for this sensor with *K*_d_ = 0.57 pM.

The AP-GQD-MIP sensor detected the original epitope within a concentration range of 0.1–10 μM, achieving an LOD of 0.1 μM experimentally. On the other hand, AP-GQD-AuNP-MIP allowed for detection over a wider concentration range (0.01–1.0 μM) with 10 times lower LOD (0.01 μM). In our study, LOD denotes the minimum discernible concentration of the target substance, detected via the sensor experimentally. The hybrid nanocomposite MIP layer, incorporating AuNPs and GQDs, exhibited superior sensitivity. The filling of cavities on the sensor surface by the analyte led to a noticeable increase in signal suppression. Figure 5A depicts the SWV voltammograms of AP-GQD-AuNP-MIP at various concentrations, with peak values around 0.2 V. As the epitope concentration increased, the cavities on the sensor surface became saturated, resulting in a significant decrease of the corresponding electrochemical signal with a linear range of 0.1–0.8 μM (Figure 5B inset). Similar results were observed for AP-GQD-AuNP, as shown in Figure S12A,B.

Binding assays were implemented to investigate the epitope’s rebinding. The same methodology was also used for the biomarker detections. The AP-GQD-MIP and AP-GQD-AuNP-MIP sensors enabled the determination of cTnI protein in concentration ranges of 0.005–50 ng mL^–1^ and 0.0005–50 ng mL^–1^, respectively (Figures 5C and S12C). The respective sensors attained LODs of 5.0 pg mL^–1^ and 0.5 pg mL^–1^. The AP-GQD-AuNP-MIP sensor showed a linear range from 1 to 10 pg mL^–1^, as shown in Figure 5D (inset), whereas the AP-GQD-MIP sensor showed a linear range (Figure S12D, inset) from 0.1 to 20 ng mL^–1^. The only difference in the composition of these two sensors is the incorporation of AuNPs into the polymeric film. However, the effect on the cTnI detection limit is noticeable, indicating that AP-GQD-AuNP-MIP has an LOD 100 times lower than that of AP-GQD-MIP. The binding behavior of the MIPs was investigated by analyzing the sensor data using three different binding isotherms: Langmuir, Freundlich, and Langmuir–Freundlich. These isotherms provide insights into the homogeneity or heterogeneity of MIP cavities.^43^ The Langmuir–Freundlich (LF) model provided the best fit for both sensors, yielding the highest *R*^2^ value. This indicates that sensors possess a combination of homogeneous and heterogeneous binding sites on the nanocomposite recognition layer (Figures 5D and S12D). Using eq S3, the dissociation constants (*K*_D_) were determined as 1.98 and 0.57 pM for AP-GQD-MIP and AP-GQD-AuNP-MIP sensors, respectively. The smaller the dissociation constant, the larger the affinity. It is noteworthy that the threshold level of the cTnI biomarker was 40 pg mL^–1^, and higher amounts in human blood are associated with increased CVD risk. Considering this, the AP-GQD-MIP sensor exhibited 8 times higher sensitivity, whereas the AP-GQD-AuNP-MIP sensor demonstrated significantly improved performance due to the synergic effects of nanomaterials by enabling the detection of concentrations 80-fold lower than the threshold. These results indicate excellent sensitivity compared to other reported protein detection assays based on epitope-mediated MIP sensors.^16,26,44−47^

### 3.7 Selectivity, Cross-Reactivity, and Stability

**Figure 6:**
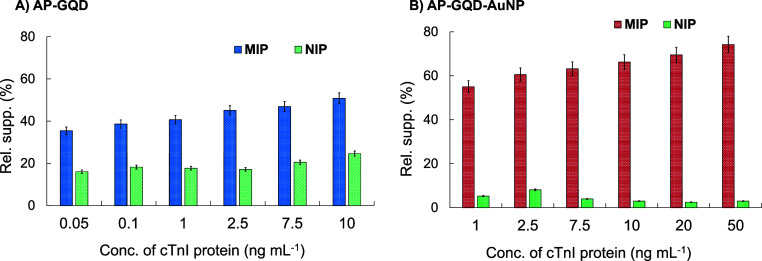
Selectivity studies with the cTnI rebinding assay. (A) AP-GQD-MIP vs NIP and (B) AP-GQD-AuNP-MIP vs NIP.

The selectivity of both sensors was evaluated by conducting rebinding assays using NIPs. The NIPs were synthesized without the Cys-epitope template, resulting in a nonselective polymer layer lacking template-specific cavities. The preparation conditions or the nonselective counterparts of both sensors were maintained exactly the same. The rebinding signals obtained from the MIPs and NIPs were averaged, and the resulting ratio (MIP vs NIP) was calculated as the IF. The AP-GQD-MIP sensor exhibited an IF of 2.2. In contrast, the AP-GQD-AuNP-MIP sensor demonstrated an IF of 15 (Figure 6), indicating significantly improved performance in establishing a reliable POCT tool with desirable selectivity.^48^

**Figure 7:**
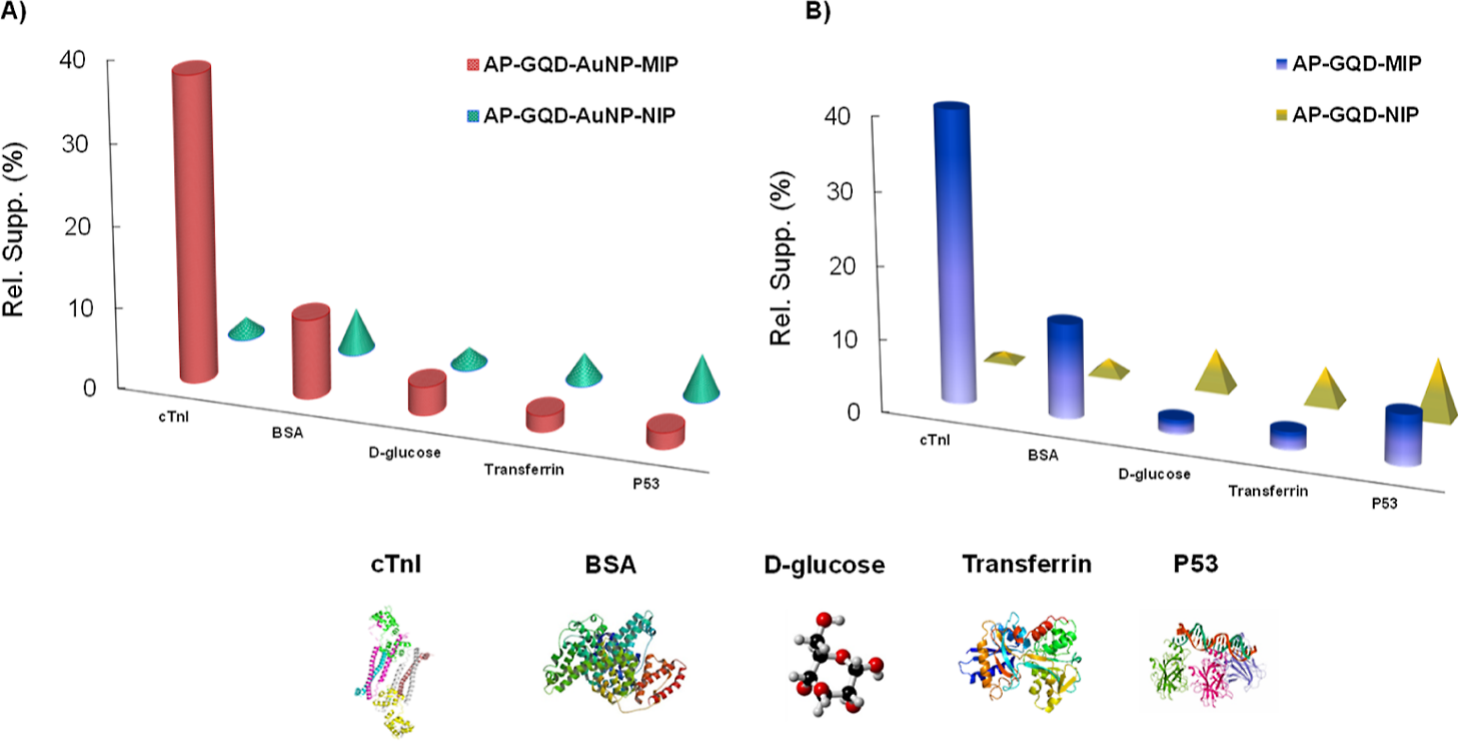
Cross-reactivity of AP-GQD-AuNP-MIP (A) and AP-GQD-MIP (B) compared to their NIPs. The concentration of all cross-reactants was 100 ng mL^–1^, and the incubation period was 30 min. The protein structures were taken from the Protein Data Bank.

The specificity of electro-MIPs was evaluated by examining their cross-reactivity profiles with reference molecules. Both sensors’ binding interactions with nonspecific proteins and small molecules were tested at a fixed concentration of 100 ng mL^–1^. The high concentration was chosen to account for the presence of various cross-reactants in clinical samples that could potentially interfere with MIP cavities. After incubating each analyte with the MIP and NIP sensors for 30 min, the electrochemical signals were recorded using the SWV technique. MIPs exhibited high specificity and minimal cross-reactivity compared to their corresponding NIPs. The AP-GQD-AuNP-MIP sensor (Figure 7A) showed superior specificity toward cTnI, indicated by lower sensing signals with all cross-reactants compared to the AP-GQD-MIP sensor (Figure 7B). This could be attributed to the AP-GQD-MIP sensor having fewer binding sites and/or less efficient binding cavities. The inclusion of AuNPs and GQDs in the nanocomposite sensor enhanced selectivity and specificity toward cTnI, likely due to the well-ordered distribution of nanomaterials near the sensor surface cavities, improving protein recognition performance. The NIP cross-reactivity results highlighted the significant influence of choosing an appropriate template in MIP synthesis, as the NIP sensors exhibited the lowest specificity.

**Figure 8:**
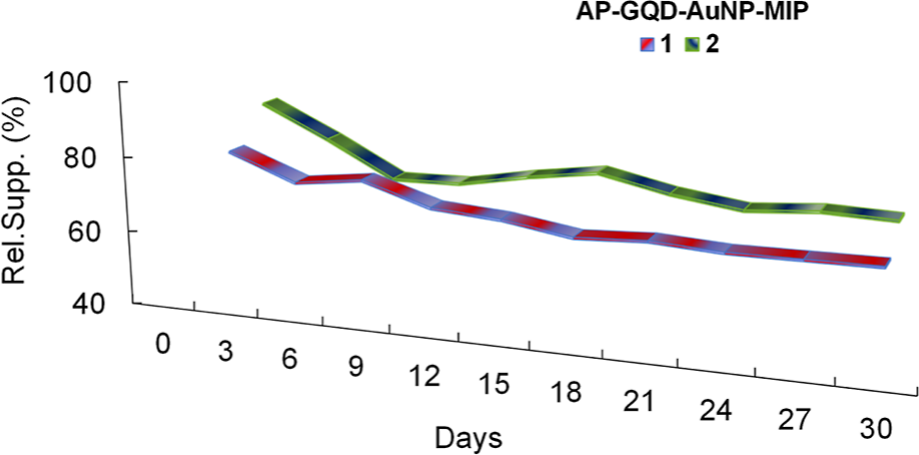
Stability test of the AP-GQD-AuNP-MIP sensor.

The AP-GQD-AuNP-MIP biosensor exhibited the best performance and underwent a critical stability test over 30 days (Figure 8). The test was performed in duplicate with a concentration of 10 ng mL^–1^ cTnI protein.

### 3.8 cTnI Detection in Human Serum

**Figure 9:**
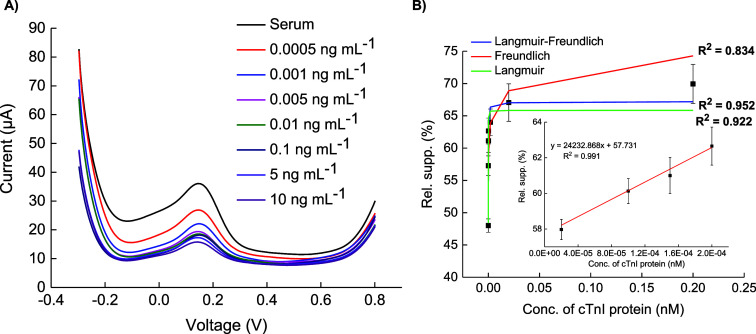
(A) AP-GQD-AuNP-MIP sensor to detect cTnI protein in human serum with a LOD of 0.5 pg mL^–1^ and (B) its corresponding binding isotherm where best fit was revealed to be the Langmuir–Freundlich (LF) model, indicating heterohomogeneous binding sites (*K*_d_ = 1.96 fM), (B) Inset graph: linear regression fit with *R*^2^ = 0.991 (*n* = 6).

To assess the clinical applicability of the developed sensors, protein detection was also performed in human serum. cTnI samples, prepared in 50% human serum, were tested using the two sensors. The RSS was calculated for each concentration of the protein with respect to the serum signal (eq S2). The nanocomposite sensor, consisting of GQDs, AuNPs, and MIPs, exhibited a detection range for cTnI from 0.0005 to 10 ng mL^–1^, with an LOD of 0.5 pg mL^–1^ (Figure 9A). The rebinding model analysis showed that LF was the best fit, yielding a *K*_D_ value of 1.96 fM (Figure 9B). The serum assay’s linearity fit (inset Figure 9B) showed a concentration range of 1–10 pg mL^–1^, which is below the cTnI threshold level. Although this would allow monitoring the healthy individuals with high precision as well as the patients, the linear response range of the sensor should be further improved for highly accurate monitoring of AMI patients. This may be achieved by multifactorial optimization of the sensor which could provide a more holistic understanding of the system.

The SWV data of cTnI detection was also fitted into the Hill eq (eq S5),^49^ as mentioned in Supporting Information. This analysis revealed the *K*_d_ of 8.7 pM which is higher than the Langmuir–Freundlich fit; however, the value is at a low nanomolar range, indicating acceptable affinity of the AP-GQD-AuNP-MIP sensor toward cTnI in serum media (Figure S13). The hill coefficient (*n*) is 0.3, which infers that negative cooperativity exists between the receptor and protein and there is competition between the binding sites. Despite the substantial decrease in current signals compared to the assays in the buffer, the high affinity and sensitivity of the sensors demonstrated their capability for ultrasensitive protein detection, even in a complex matrix-like human serum.

Table 1 provides a comprehensive comparison between the current AP-GQD-AuNP-MIP electrochemical biosensor and similar studies focusing on biosensors for cardiac biomarker detection. Notably, the analytical performance of the AP-GQD-AuNP-MIP sensor was evaluated using SWV, a departure from the commonly employed differential pulse voltammetry (DPV) in other reports. A noteworthy advantage of SWV, as opposed to DPV, is the significantly reduced measurement duration. The efficiency of SWV is highlighted by its ability to accomplish experiments that would typically take 3 min using differential pulse techniques in a matter of mere seconds.^50^ Hence, the detection time required for cTnI in human serum using the AP-GQD-AuNP-MIP sensor is only 18 min. This rapid detection time positions the sensor as highly valuable for potential future POCT devices.

**Table 1:** Comparison of Current Biosensors for the Detection of Cardiac Biomarkers^a^

target biomarker	electrode/sensor type	materials	method	investigation range	LOD	reference
cTnT	SPCE (disposable)	PANI	DPV	0.1–8.0 pg mL^–1^	0.04 pg mL^–1^	(53)
cTnT	SPCE (disposable)	PANI	DPV	0.02–0.09 ng mL^–1^	0.008 ng mL^–1^	(54)
cTnI	SPGE (disposable)	anti-cTnI/AuNP@GQD	CV, SWV, EIS and Amperometry	1–1000 pg mL^–1^	0.5 pg mL^–1^	(5)
cTnI	GCE (reusable)	ZnONPs/MIP/Apt	EIS	1.25 × 10^–5^–8.25 μg mL^–1^	2.61 × 10^–5^ μg mL^–1^	(55)
cTnI	GCE (reusable)	*o*-AP	EIS	1.195–119.5 ng mL^–1^	0.65 ng mL^–1^	(56)
cTnI	GCE (reusable)	PPy	DPV	0.01–5.0 ng mL^–1^	0.0005 ng mL^–1^	(52)
cTnI	GCE (reusable)	MAA	DPV	0.005–60 ng mL^–1^	0.0008 ng mL^–1^	(51)
cTnI	GCE (reusable)	MNPs/Tro6-cTnI-Tro4/BPB@Ti_3_C_2_	Chronoamperometry	200 fg mL^–1^ – 250 ng mL^–1^	20 fg mL^–1^	(57)
cTnI	SPR sensor chip	NanoMIPs	SPR	0.78–50 ng mL^–1^	0.52 ng mL^–1^	(58)
cTnI	gold (reusable)	Cys-epi/o-AP/GQD/AuNP	SWV	0.0005–10 ng mL^–1^	0.5 pg mL^–1^	current work

a Abbreviations: SPCE—screen printed carbon electrode, GCE—glass carbon electrode, SPE—screen printed electrode, Au—gold, cTnI—cardiac troponin I, Myo—myoglobin, cTnT—cardiac troponin T, EIS—electrochemical impedance spectroscopy; DPV—differential pulse voltammetry; CV—cyclic voltammetry; SWV—square wave voltammetry, MIP—molecularly imprinted polymers, AuNPs—gold nanoparticles, GQDs—graphene quantum dots, rGO—reduced graphene oxide, ZnONPs—zinc oxide nanoparticles, Cys-epi—cysteine epitope, PANI—poly(aniline), *o*-AP—*ortho*-aminophenol, *o*-PD—*o*-phenylenediamine, PPY—poly(pyrrole), Apt—aptamer, AAM—acrylamide, MAA—methacrylic acid, anti—antibody, MNP—magnetic nanoparticles, Tro6/4-cTnI aptamers, BPB—bromophenol blue, Ti_3_C_2_-Mxene (2D material), SPR—surface plasmon resonance, NanoMIPs—molecularly imprinted polymer nanoparticles. The LOD mentioned in these reported works of literature is calculated from the calibration curve (LOD = 3.3σ/S), whereas the LOD mentioned in our work is determined experimentally.

The work of Ma et al. involves the development of MIPs utilizing cTnI (whole protein) as a template along with acrylic monomers, chitosan, glutaraldehyde, and other chemical components.^51^ The process entails numerous synthesis and sensor fabrication steps, which collectively contribute to its labor-intensive nature. Moreover, their evaluation of sensor stability was limited to a relatively short 14 day period at low temperature (4 °C). In a similar vein, the research conducted by Yola and Atar showed challenges associated with the synthesis of essential two-dimensional (2D) hexagonal boron nitride due to the intricate and demanding synthesis procedures involved. Although the LOD achieved by their sensor aligns with the results of our study, the detection range exhibited by their sensor is somewhat constrained, lacking the desired extent.^52^ Phonklam et al. adopted a distinctive approach utilizing MIPs on screen-printed carbon electrodes, coupled with multiwalled carbon nanotubes modified via electrodeposition with the redox probe polymethylene blue for sensing the cTnT. This sensor demonstrated the ability to detect cTnT within the range of 0.10–8.0 pg mL^–1^ using DPV. Regrettably, this concentration range falls short of encompassing the crucial threshold limit for cardiac biomarkers, presenting a notable disadvantage. Additionally, this screen-printed electrode sensor falls under the category of disposable sensors. The process of fabricating this sensor involves intricate steps such as glutaraldehyde activation, the participation of lithium compounds, and overnight incubation, which collectively introduce a level of complexity that can be burdensome to execute effectively.^53^ Turning to the work by Karimi et al., their research presented its own challenges. For instance, the synthesis of reduced graphene oxide demanded a duration of 48 h, and the subsequent deposition onto a screen-printed electrode entailed a series of multiple steps. Such intricate and time-consuming procedures can potentially hinder the scalability and practical implementation of their approach.^54^ However, a common drawback among these sensors is their single-use design, necessitating repetitive electrode treatment steps, and is disadvantageous in terms of practicality and usability. In the study conducted by Mokhtari et al., their approach involved the synthesis of nanomaterials (particularly, zinc oxide) through a biological pathway employing leaf extract. However, this method introduces the probable variability in synthesis outcomes between different batches. Additionally, the process of incubating the aptamer-cTnI complex with the modified electrode surface is relatively lengthy, spanning an extended duration of 90 min. This extended incubation time may affect the sensor’s overall efficiency. Moreover, the steps entailed in modifying the electrode are intricate and time-consuming, potentially hindering practical application. Notably, the detection range exhibited by this sensor is somewhat limited in scope.^55^ Furthermore, the stability assessment was conducted under specific conditions—low temperature (5 °C) over a span of 20 days which might not fully capture the sensor’s performance in more varied environments. The MIP-based sensor developed by Zuo et al. presents a notable achievement in the detection of cTnI using *ortho*-aminophenol as the electroactive monomer.^56^ However, several challenges persist in the overall protein imprinting process. Notably, the TR step entails a lengthy elution period of 4 h, which is unfavorable for the sensor fabrication process. Furthermore, the sensor’s LOD exceeds the desired threshold level, and the electrochemical cell necessitates a substantial volume of 25 mL of solution for method execution. In the detection assay, the observed reduction in DPV signal lacks significant magnitude. Moreover, the stability of the sensor remains unreported, presenting a crucial aspect requiring investigation for comprehensive sensor characterization.

In addition to electrochemical MIP-based sensor strategies, cardiac biomarkers have been detected utilizing diverse transducers and novel nanocomposite materials. For instance, Khoshfetrat and Chegeni et al. have recently presented the utilization of the BPB@Ti_3_C_2_ nanocomposite to detect the cTnI biomarker.^57^ They synthesized Ti_3_C_2_ MXene nanomaterial employing bromophenol blue (BPB) to develop a sandwich-type aptasensor, MNPs/Tro6-cTnI-Tro4/BPB@Ti_3_C_2_, highlighting the extraordinary stability of the aptamers on MNPs and BPB@Ti_3_C_2_ through the strong formation of covalent bonding. This aptasensor exhibited high selectivity, sensitivity (LOD = 20 fg mL^–1^), reproducibility, and reusability. However, the introduced sandwich assay required two receptor-like molecules with complicated procedures compared to our work.

In our earlier research, we have introduced a SPR sensor utilizing nanoMIPs as epitope-based receptors, aimed at the sensitive detection of cardiac troponin I (cTnI).^58^ The nanoMIPs manufactured by the solid-phase synthesis protocol were covalently immobilized onto an SPR chip using EDC-NHS coupling chemistry. This optical sensor has allowed real-time detection of cTnI within a concentration range of 0.78–50 ng mL^–1^, with an LOD of 0.52 ng mL^–1^. Notably, the sensor exhibits high selectivity and specificity, highlighting its potential for precise biomarker detection. However, considering the threshold level (0.04 ng mL^–1^) of the cTnI biomarker, the sensitivity of this sensor must be further improved for POCT applications of AMI detection.

In striking contrast to previously documented studies, the development of our AP-GQD-AuNP-MIP sensor distinguished itself through its expeditious and less convoluted fabrication process. Notably, this sensor demonstrated an extended concentration range spanning from 0.0005 to 10 ng mL^–1^, accompanied by an excellent LOD at 0.5 pg mL^–1^. This encompassing range is of particular significance as it effectively covers the pivotal threshold for cardiac troponins, and crucially, it avoids immediate saturation, signifying a commendable feature for sensors. Furthermore, the nanocomposite sensor was subjected to a comprehensive stability assessment at room temperature over a period of 30 days. Moreover, the inherent reusability of the MIP sensor presents an attractive attribute, especially in the context of POCT tools. However, the detection of the target is indirect as it involves the usage of a redox marker solution (potassium ferrocyanide) which could pose a limiting factor when it comes to potential POCT applications. Nevertheless, the proposed MIP-based nanocomposite sensing film can also be combined with different readout systems for the development of POCT devices. The LOD mentioned in the reported literature is calculated from a calibration curve (LOD = 3.3σ/S), whereas the LOD mentioned in our work is determined experimentally. The amalgamation of these advantageous features positions our sensor as a promising advancement in the field of cardiac biomarker detection.

## 4 Conclusions

In the field of health care and medical diagnosis, developing a reliable, rapid, and highly sensitive biosensor for the detection of specific biomolecules is a significant challenge. This research work presents a strategy that combines electro-MIP and smart nanomaterials to detect the cTnI protein specifically. To simplify the imprinting process, a cysteine-modified cTnI epitope was utilized as the template. For electropolymerization, the functional monomer of 2-AP was employed, along with signal-amplifying agents including GQDs and AuNPs. The parameters such as incubation period, concentration, polymerization cycles, and TR steps were comprehensively optimized using electrochemical techniques such as SWV, CV, and EIS. Two optimized sensors, namely, AP-GQD-MIP and AP-GQD-AuNP-MIP, were characterized using various techniques, including AFM, fluorescence microscopy, contact angle analysis, and ATR-FTIR. These analyses provided a comprehensive understanding of their surface topologies, hydrophilicity features, and chemical functionalities. Both sensors exhibited sensitive detection of cTnI protein at low concentrations in the detection assay. The AP-GQD-AuNP-MIP sensor demonstrated a 10-fold lower LOD compared to the AP-GQD-MIP sensor, indicating the strong nanohybrid effects on the active sites of the MIP films. Furthermore, the AP-GQD-AuNP-MIP sensor exhibited higher sensitivity and selectivity, with reduced cross-reactivity compared to the AP-GQD-MIP sensor. The NIP films showed no specificity toward cTnI proteins, emphasizing the crucial role of the template in MIP-based biosensors. Moreover, the best-performing AP-GQD-AuNP-MIP sensor showed remarkable stability over a period of 30 days. This sensor exhibits great potential to develop a cost-effective, user-friendly, highly sensitive biosensor with a synthetic receptor for specific detection of cTnI. In future work, one of the goals will be developing an alternative strategy for direct target detection without the need for redox marker solutions which would be more suitable for POCT devices.

## Supporting Information Available

Experimental parameters used for electrochemical techniques; calculation of RSS and dissociation constant; characterization; optimizations of template concentration and TR; electropolymerization of 2-AP; characterization of the molecular imprinting process; and cTnI epitope rebinding assay (PDF)
