# Supplementary material for: Cardiac Troponin I-Responsive Nanocomposite Materials for Voltammetric Monitoring of Acute Myocardial Infarction
Source: ACS Omega. 2024 Jul 6;9(28):30737–50. doi: 10.1021/acsomega.4c03252 (PMC11256321; doi:10.1021/acsomega.4c03252)
Supplement: Supplementary file 1 — ao4c03252_si_001.pdf [file ao4c03252_si_001.pdf]

Supporting Information

Cardiac Troponin I-responsive Nanocomposite Materials for Voltammetric Monitoring of Acute Myocardial Infarction

Gauri Kishore Hasabnis[a,b], *and Zeynep Altintas*[a,b,c*]

[a]Institute of Chemistry, Faculty of Natural Sciences and Maths, Technical University of Berlin, Straße des 17. Juni 124, 10623 Berlin, Germany

[b]Institute of Materials Science, Faculty of Engineering, Kiel University, 24143 Kiel, Germany

[c]Kiel Nano, Surface and Interface Science (KiNSIS), Kiel University, 24118 Kiel, Germany

* Corresponding author:

Prof. Dr. Z. Altintas

zeynep.altintas@tu-berlin.de | zeynep.altintas@tf.uni-kiel.de

EXPERIMENTAL DETAILS

Reagents and chemicals

Gold wires (Goodfellow) with a diameter of 0.5 mm were used as the working electrode (WE). Potassium hydroxide (≥ 85%, KOH, MW: 56.11 g/mol, Carl Roth) was used to clean the gold wires, which were afterward stored in sulfuric acid (≥ 95-98%, H_2SO_4, MW: 98.08 g/mol, Carl Roth). Prior to each experiment, concentrated nitric acid (≥ 65%, HNO_3, MW: 63.01 g/mol, Carl Roth) was used to activate the surface of the wires. For the electrochemical measurements, potassium hexaferricyanide (≥ 99%, $K_3[Fe(CN)]_6$, MW: 329.26 g/mol, Carl Roth) was used as a redox marker in cyclic voltammetry (CV), square wave voltammetry (SWV) and electrochemical impedance spectroscopy (EIS) techniques. A supporting salt of potassium chloride (≥ 99.9%, KCl, MW: 74.55 g/mol, VWR Chemicals) was added to enhance the conductivity of the redox probe. Double-distilled ultrapure water was obtained from a Millipore Direct-Q 3 UV (Millipore, Germany). Graphene quantum dots (GQDs, blue luminescent, 1 mg/mL in H_2O, Sigma-Aldrich) and gold nanoparticles (AuNPs, 7×10^{13} NPs/mL, in-house synthesis) were used to amplify the detection signals and increase the sensitivity of the biosensors. For AuNP synthesis, gold (III) chloride trihydrate (Tetrachloroauric acid, $HAuCl_4$, M.W.: 393.83 g mol^{-1}, Sigma Aldrich); Potassium carbonate: (K_2CO_3, M.W.: 138.21 g mol^{-1}, Sigma Aldrich); Tannic acid ($C_{76}H_{52}O_{46}$, M.W.: 1701.19 g mol^{-1}, Sigma Aldrich) and sodium citrate tribasic dehydrate ($C_6H_5Na_3O_7.{}_2H_2O$, M.W.: 294.1 g mol^{-1}, Sigma Aldrich) were used.

The functional monomer 2-aminophenol (2-AP, ≥99%, C_6H_7NO, MW: 109.13 g/mol, Alfa Aesar) was used for producing sensor films. Phosphate buffer saline (PBS, pH: 7.2-7.6, Sigma-Aldrich) served as the pre-electropolymerization solution, as well as the medium for template removal and rebinding assays. PBS buffer was prepared by dissolving one tablet in 200 mL of deionized water, resulting in a pH of 7.4 at 25°C with concentrations of 0.01 M phosphate buffer, 0.0027 M potassium chloride, and 0.137 M sodium chloride. The targets in the detection assays were cardiac troponin I protein (cTnI, SDS-PAGE, MW: 52 kDa, Merck-Millipore) and in-house-synthesized cTnI-derived original peptide (ISASRKLQLK). Cysteine modified peptides (CISASRKLQLK, Cys-epitope) were used as templates for molecularly imprinted polymer (MIP) films, while non-imprinted polymers (NIP) films were synthesized without this peptide. For template dissolution, phosphate buffer saline with tris(2-carboxyethyl) phosphine hydrochloride (PBS-TCEP, pH: 7.5) was prepared using PBS tablet, TCEP, and sodium hydroxide (NaOH). Bovine serum albumin (BSA, ≥98%,

chromatographically purified, MW: 66 kDa, Sigma-Aldrich), transferrin (≥98%, MW: 80 kDa, Sigma-Aldrich), P53 protein (SDS-PAGE, MW: 49 kDa, Sigma-Aldrich), and glucose (≥99.5%, MW: 180.16 g/mol, Sigma-Aldrich) were employed as cross-reactants for specificity studies. Gold-coated silicon wafers (Plano GmbH, Wetzler, Germany) were utilized as chips to mimic gold wires for surface characterization studies using atomic force microscopy (AFM). For contact angle measurements and fluorescence microscopy, screen-printed gold electrodes (SPGEs; DS220AT, Metrohm, Germany) were employed as flat surfaces. The MIP film on SPGEs was synthesized using the Metrohm DropSens connector with identical parameters to those used for gold wires.

Working electrode preparation

The gold wires underwent two rounds of boiling in a 2.5 M KOH solution for 4 hours and were subsequently stored in concentrated sulfuric acid (H_2SO_4). Prior to each experiment, the wires were immersed in concentrated nitric acid (HNO_3) for 10 min, rinsed with Millipore water, and carefully dried using nitrogen (N_2) gas. For AFM samples, gold-coated silicon wafer chips replaced the gold wires as the working electrode. These chips were cleaned by immersion in an acidic piranha solution (sulfuric acid [H_2SO_4] and hydrogen peroxide [H_2O_2], x:y ratio, 3:1) for 5 min and then dried under a moderate flow of N_2 gas.

Electrochemical measurements

Electrochemical measurements were performed in a 2.0 mL electrochemical cell using a three-electrode system. Gold and platinum wires served as the WE and counter electrode (CE), respectively, while a silver electrode (Ag/AgCl) functioned as the reference electrode (RE). The PalmSens4 workstation (Belltec, Germany) was used for all electrochemical protocols. CV, SWV and EIS measurements were carried out in a solution containing 10 mM $K_3[Fe(CN)]_6$ and 0.1 M KCl at room temperature. For CV, a potential range of -0.2 to 0.8 V and a scan rate of 0.05 V s^{-1} were employed. SWV measurements were performed with potentials ranging from -0.3 to 0.8 V, an amplitude of 0.05 V, and a frequency of 5 or 10 Hz. Prior to each measurement, the cell was cleaned by rinsing it three times with double-distilled water and dried using a gentle flow of N_2 gas. Each measurement was repeated at least three times to ensure reproducibility and reliability of the results. Table S1 shows the final parameters used in electrochemical characterization, electropolymerization, and involved in the template removal process.

Table S1: List of parameters of electrochemical methods.

Parameters of electrochemical methods					
Cyclic voltammetry (CV)		Square wave voltammetry (SWV)		Electrochemical impedance spectroscopy (EIS)	
E condition	0 V	E condition	0 V	E condition	0 V
t condition	0 s	t condition	0 s	t condition	0 s
E begin	0.6 V	E begin	-0.2 V	E dc	0.0 V
E vertex	0.6 V	E end	0.8 V	E ac	0.01 V
E vertex 2	-0.2 V	E step	0.003 V	Frequency type	Scan
E step	0.004 V	Amplitude	0.05 V	n frequencies	48 = 10 /dec
Scan rate	0.1 V s^{-1}	Frequency	10 Hz	Max. frequency	50000 Hz
No. of scan	3	No. of scan	3	Min. frequency	1.0 Hz

Electropolymerization		Template removal	
Cyclic voltammetry (CV)		Multistep amperometry (MA)	
E condition	0 V	E condition	0 V
t condition	0 s	t condition	0 s
E begin	0.0 V	t intervals	0.002 s
E vertex	0.0 V	Cycles	1
E vertex 2	0.8 V	No. of steps	1
E step	0.004 V	E level 1	1.2 V
Scan rate	0.05 V s^{-1}	t 1	30 s
No. of scan	10	No. of Scan	3

Synthesis of gold nanoparticles (AuNPs)

For the synthesis of AuNPs, the strategy described by Piella et al.[1] was employed. In a three-necked round bottom flask, 0.1 mL of tannic acid (2.5 mM) was stirred with 1 mL of potassium carbonate (150 mM) and 150 mL of sodium citrate (2.2 mM) to form a reducing mixture. After the mixture attained 70°C, 1 mL of tetrachloroauric acid (25 mM) was injected. Within a couple of seconds, the solution turned blackish grey, subsequently orange-red, and the AuNPs were obtained. The resultant particle (~3.5 nm) concentration was 7×10^{13} NPs mL^{-1}.[2]

List of Equations

Equation S1. Relative Signal Suppression (RSS %) for SAM and TR conditions

$$RSS[\%] = \left(\frac{Bare_{peak\ current} - SAM\ or\ TR_{peak\ current}}{Bare_{peak\ current}}\right) \times 100 \qquad (S1)$$

Equation S2. Relative Signal Suppression (RSS %) for target detection

$$RSS[\%] = \left(\frac{TR\ or\ Serum_{peak\ current} - Target_{peak\ current}}{TR\ or\ Serum_{peak\ current}}\right) \times 100 \qquad (S2)$$

Equation S3. Dissociation constant K_D can be calculated [3] using the following equation

$$logK_D = mloga \qquad (S3)$$

where *a* corresponds to the mean association constant and *m* represents the heterogeneity index.

Equation S4. The apparent charge transfer rate constant (K_{app}) was calculated from the R_{ct} values in the presence of $[Fe(CN)6]^{3-/4-}$ using the following equation.

$$K_{app} = \frac{R\,T}{n^2 F^2 A\;C\;R_{ct}} \qquad (S4)$$

where R is the ideal gas constant (8.314 J K^{-1} mol^{-1});

T is the absolute temperature (298 K);

n is the number of transferred electrons (1);

F is Faraday number (96485.3 C mol^{-1});

A is the geometric surface area of the electrode (0.2395 cm^2);

and C is the concentration of the redox probe (10 mM)

Equation S5. Hill equation describing the cooperativity of the binding interactions between receptor and protein. The affinity of the MIP sensor towards the cTnI target was investigated with varying concentrations of target protein by employing the SWV technique[4].

$$[A] + n[B] \underset{K_d}{\overset{K_a}{\rightleftharpoons}} [AB_n]_{\text{COMPLEX}}$$

Where A – Target, B – Ligand, n – number of binding sites, K_a – Association constant, K_d – Dissociation constant

To plot the Hill equation the logarithm is taken and binding data is fitted to linear fit in the form of y = mx + c

$$log\left(\frac{Y}{1-Y}\right) = log\left(\frac{1}{K_d}\right) + n\;log\;(cTnI\;conc.) \qquad (S5)$$

Where $Y = \frac{\Delta\,SWV_{\,peak}}{\Delta SWV_{peak,max}}$, $\Delta\,SWV_{\,peak}$, $\Delta\,SWV_{\,peak\,max}$ stands for change in SWV peak and maximum change in SWV peak; n -Hill coefficient

Supporting Figures

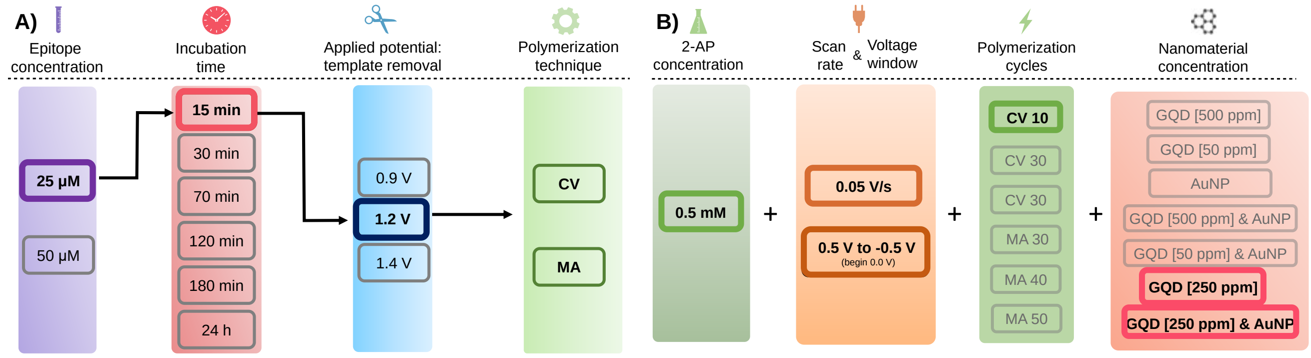

***Abbreviation**: CV-Cyclic voltammetry, MA-Multistep amperometry, GQD-Graphene quantum dots, AuNP-Gold nanoparticles (7×10^{13} NPs mL^{-1})

Figure S1: The strategic mapping for conducting optimization studies, A) Optimization of template concentration, its incubation time, and template removal conditions, B) Optimization of 2-AP monomer as well as nanomaterials concentration (with optimized template and monomer parameters).

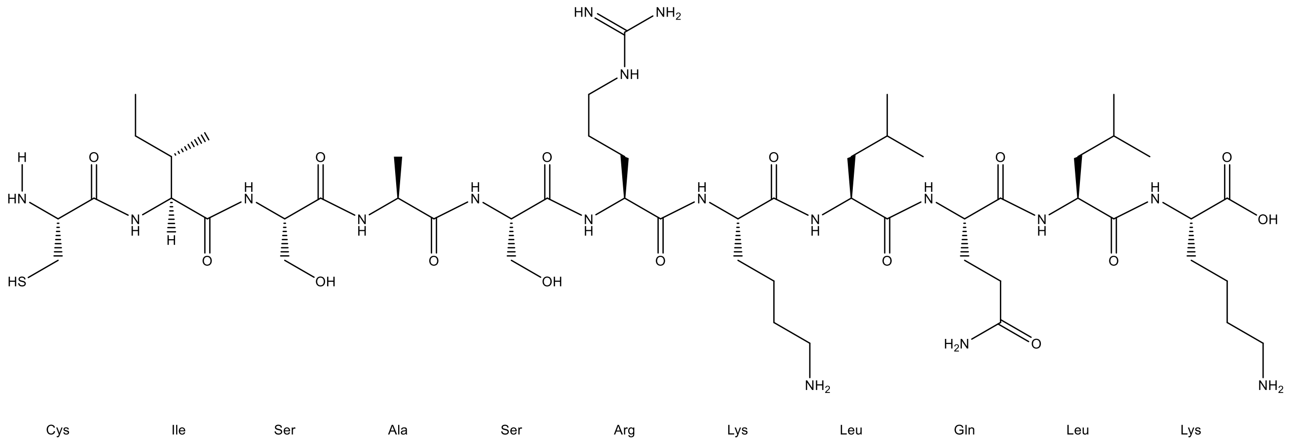

Figure S2. The peptide chain of cysteine modified cTnI epitope (Cys-epitope).

Epitopes are amino acid chains within proteins that are typically found on the protein's surface and are accessible for binding to their respective receptors. For sensor preparation, carefully selected epitopes can be chemically modified and immobilized on the sensor surface prior to the imprinting process. Modifying the epitope with a cysteine group is particularly advantageous, as cysteine contains functional groups like -SH and -NH_2 that exhibit a strong affinity for gold, enabling the formation of a SAM [5,6]. Therefore, during the synthesis of the cTnI epitope, L-cysteine was attached to the end of the epitope to facilitate template modification.

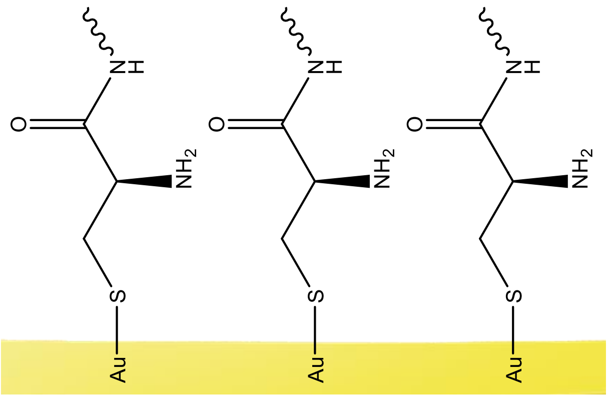

Figure S3. Attachment of Cys-epitope to gold (Au) surface (SAM).

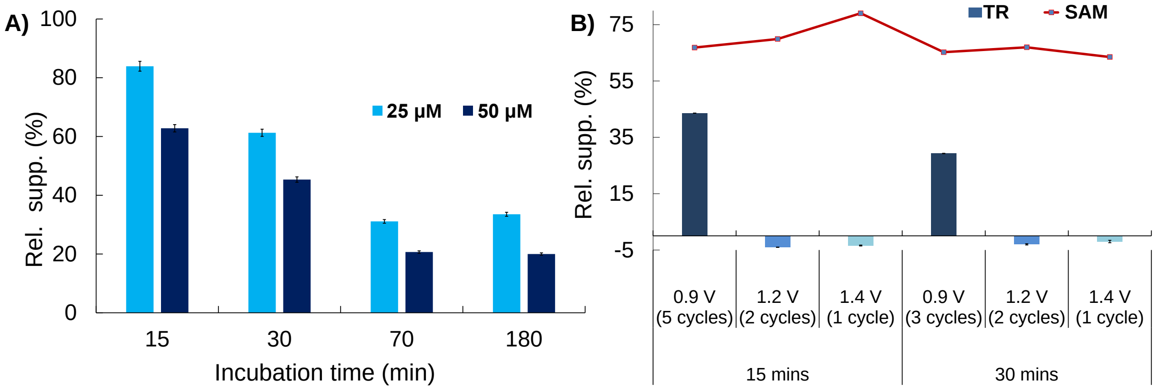

Figure S4. A) The optimization of concentration and incubation period of template (Cys-epitope) adsorption and **B**) the optimization of voltage for template removal.

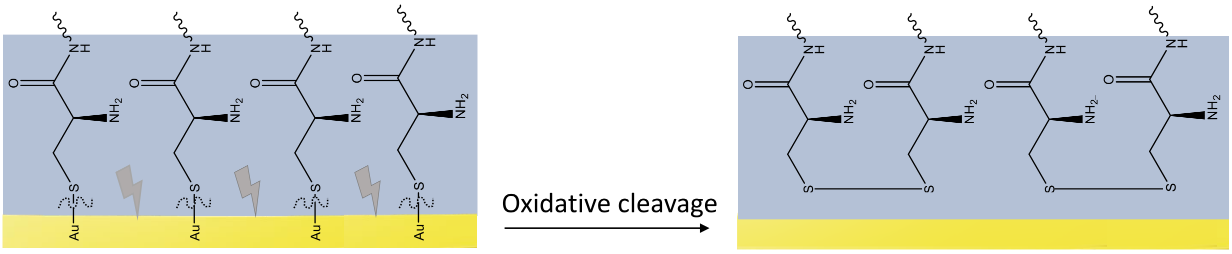

Figure S5. Plausible mechanism of electrooxidation pathway of cysteine for removal of the template.

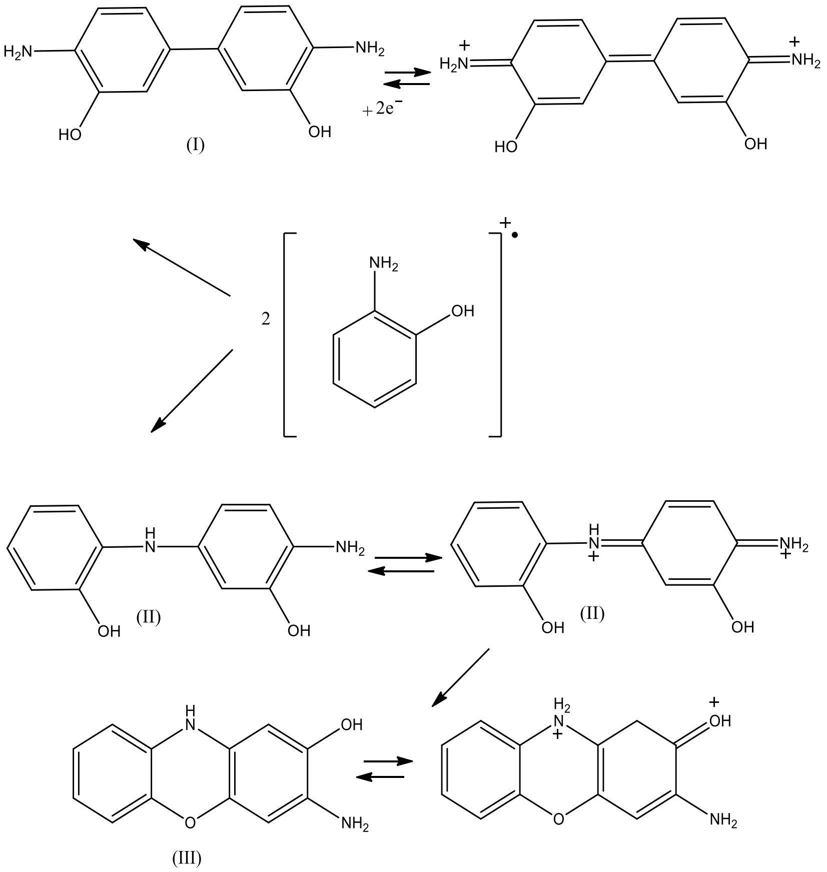

Figure S6. Plausible mechanism of PAP formation [7].

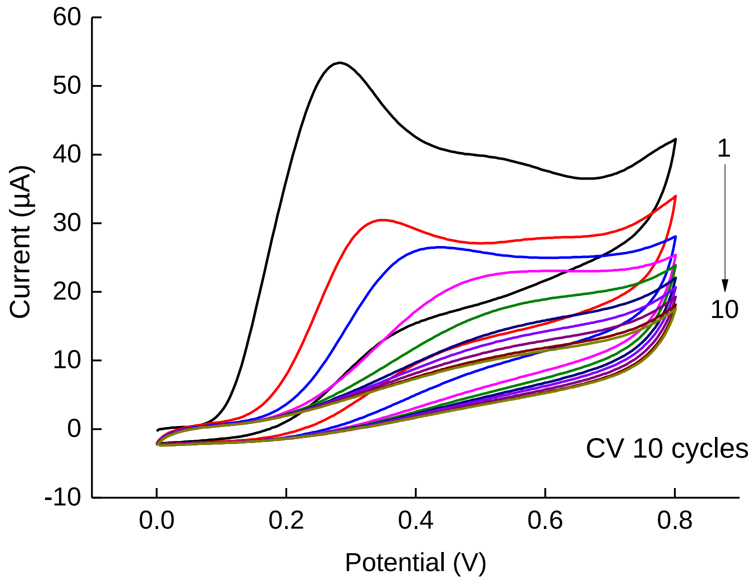

Figure S7. The electropolymerization of functional monomer (2-AP, 0.5 mM) (scan rate: 50 mV s^{-1}).

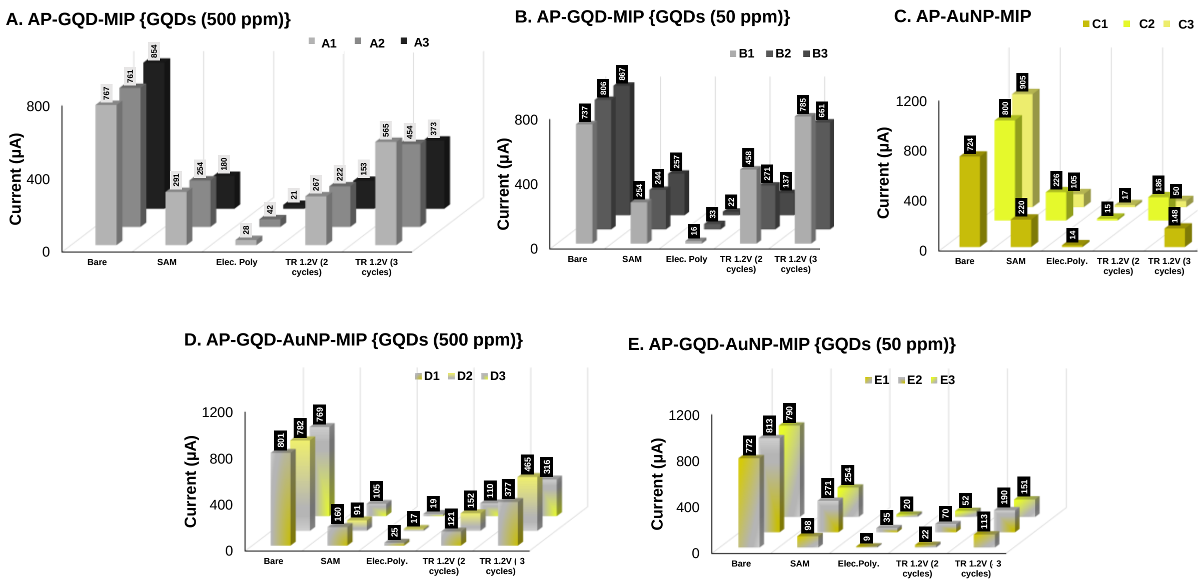

Figure S8A. Optimization results of different concentrations and compositions of GQD and AuNPs sensors, and combinations of their nanocomposite sensors. Each sensor type was examined with 3 replicas.

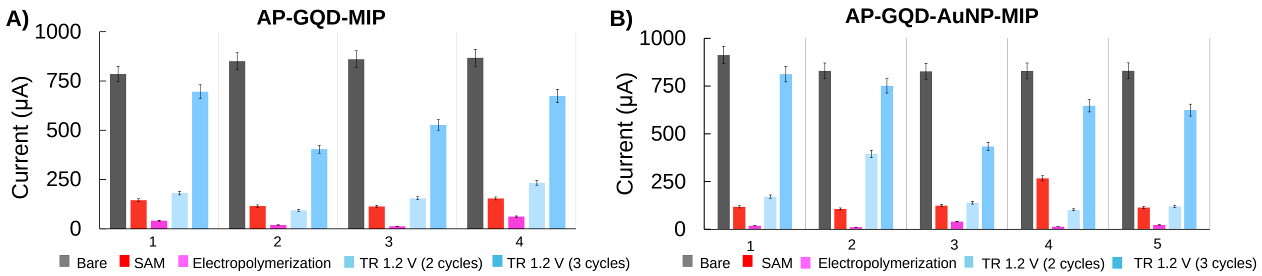

Figure S8B. Optimization results for **A)** AP-GQD-MIP and **B)** AP-GQD-AuNP-MIP (n = 3).

Table S2: The apparent charge transfer rate constant (K_{app}) extracted from the EIS data from Figure 1C and Figure S9C obtained for modified electrodes.

Electrode	R_{ct} (Ω)	K_{app} (cm s^{-1}) × 10^{-5}
Au/Cys-epitope	1259	8.96
Au/Cys-epitope/AP-GQD-MIP	14240	0.79
Au/AP-GQD-MIP (TR)	5019	2.25
Au/Cys-epitope	1455	7.75

Au/Cys-epitope/AP-GQD-AuNP-MIP	24100	0.47
Au/AP-GQD-AuNP-MIP (TR)	7236	1.56

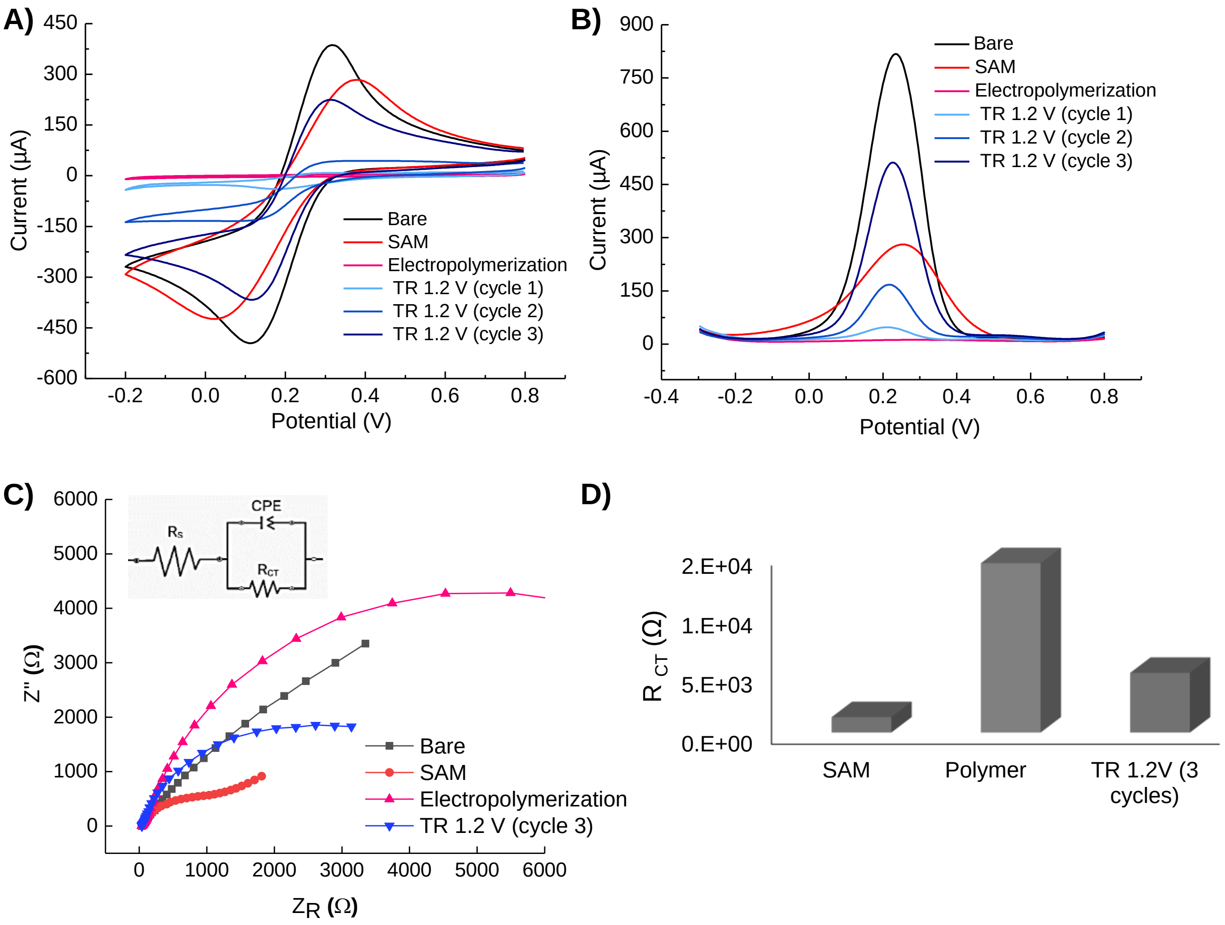

Figure S9. Electrochemical characterization of AP-GQD-MIP sensor with **A)** CV, **B)** SWV and **C)** EIS (Nyquist plot) using a redox marker solution. **D)** The R_{ct} at individual fabrication steps where the highest resistance indicated the formation of AP-GQD-MIP polymeric film (R_{ct} for SAM- 1259 Ω, polymer- 14240 Ω and TR- 5019 Ω).

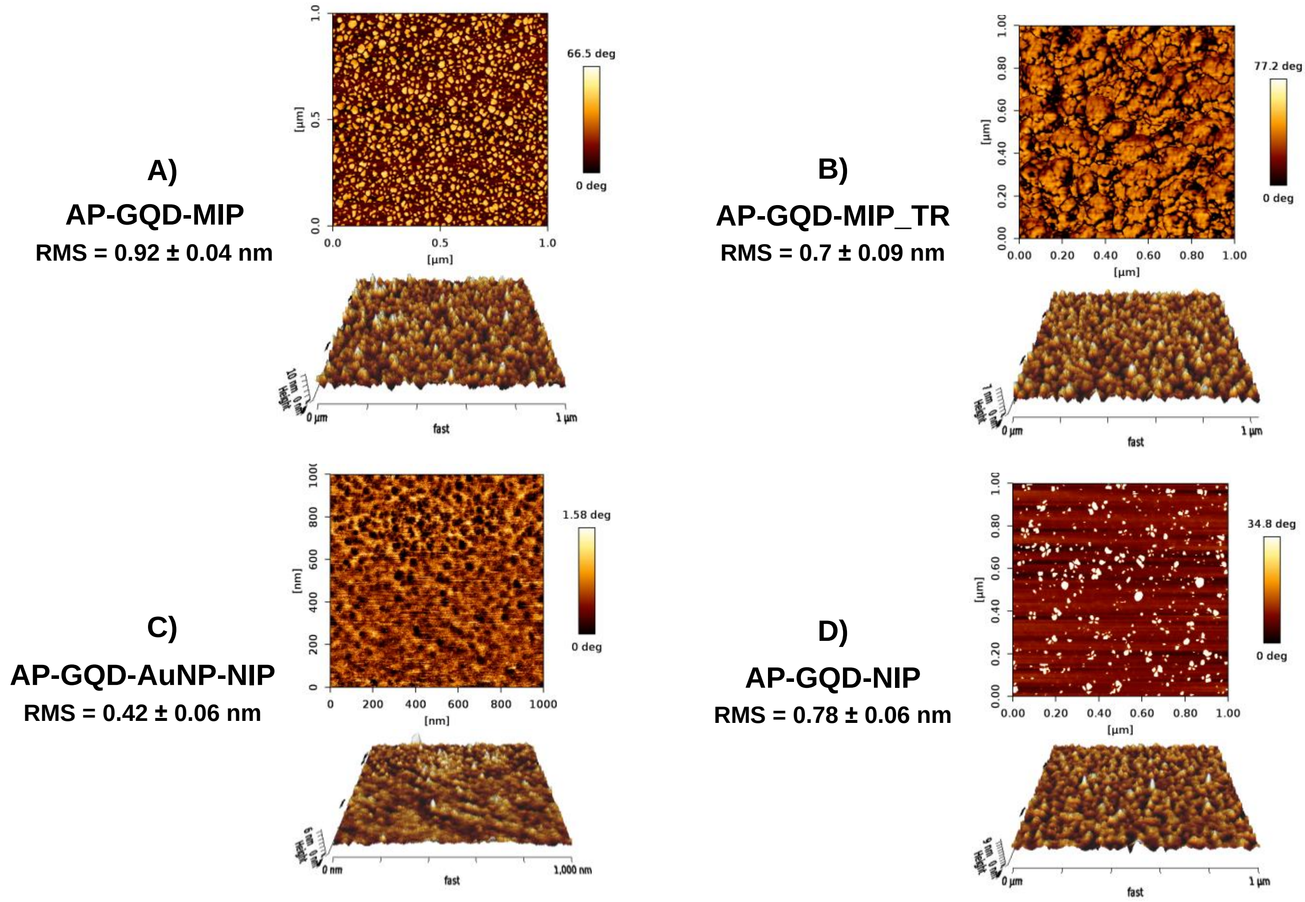

Figure S10. The AFM phase and 3D height images of **A**) AP-GQD-MIP, **B**) AP-GQD-MIP_TR, **C**) AP-GQD-AuNP-NIP, and **D**) AP-GQD-NIP.

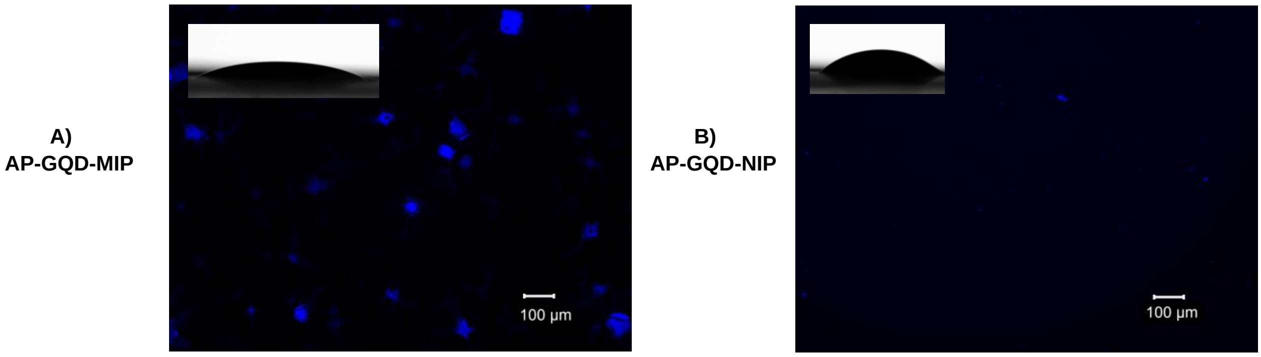

Figure S11. Fluorescence microscopic images with corresponding contact angles of **A**) AP-GQD-MIP (CA: 26.1º), and **B**) AP-GQD-NIP (CA: 41.6º).

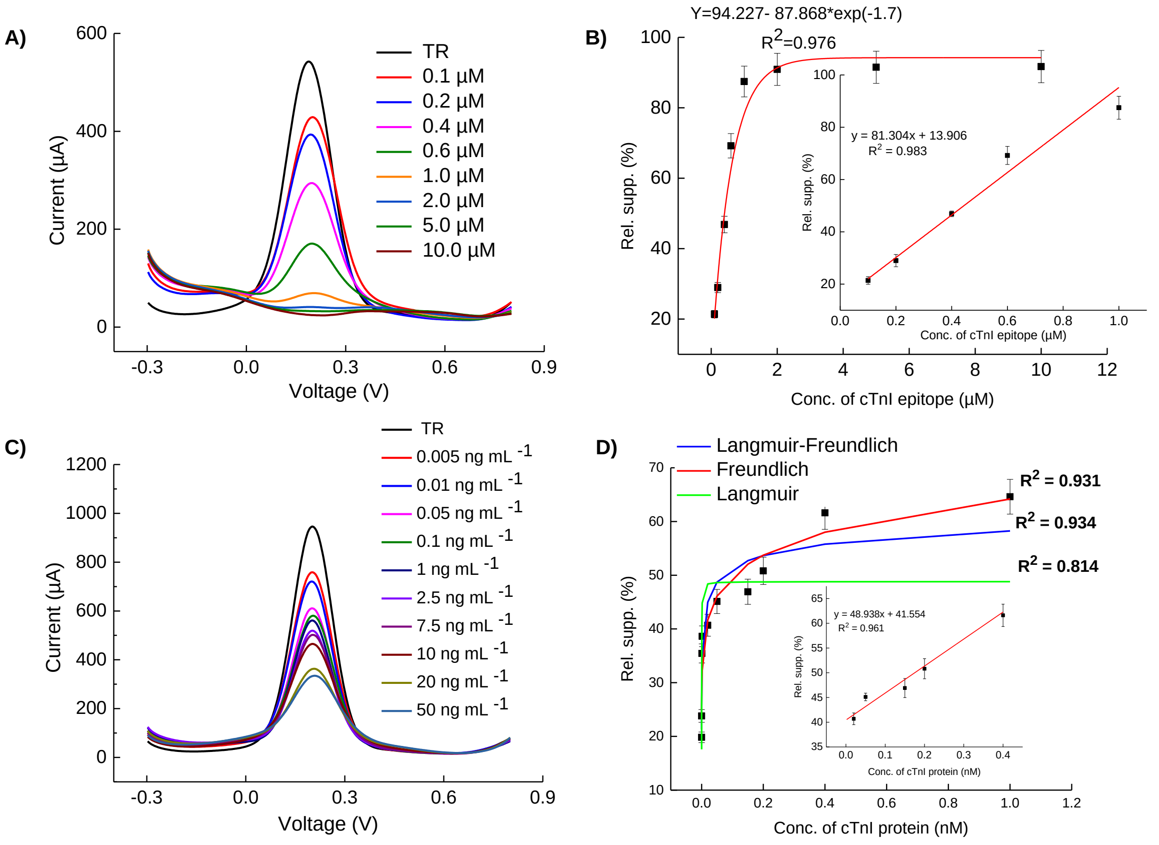

Figure S12. The cTnI epitope rebinding assay **A)**, its concentration dependency revealing linear range 0.1-1 µM (n=6) **B)**, cTnI protein detection **C),** and corresponding binding isotherm with linear regression fit (inset) **D)** of AP-GQD-MIP (n=6). Langmuir-Freundlich model (blue curve) was the best fit for this sensor with K_D= 1.98 pM.

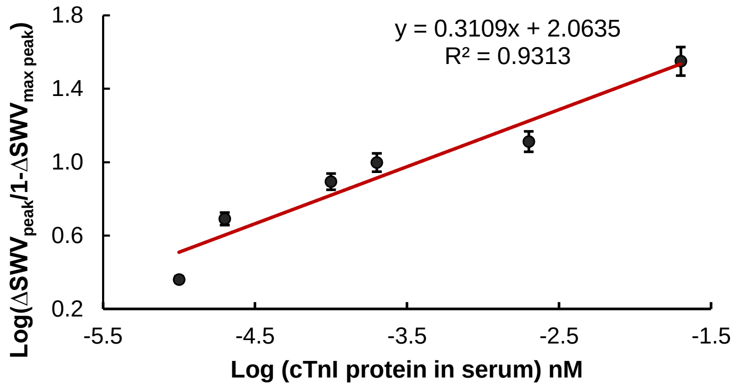

Figure S13. Hill equation plot of log (ΔSWV $_{peak}$/1-ΔSWV $_{max\ peak}$) vs. log (cTnI protein in serum), where Hill coefficient n=0.3 K_d = 8.7 pM for AP-GQD-AuNP-MIP.

REFERENCES

(1) Piella, J.; Bastus, N. G.; Puntes, V. Size-Controlled Synthesis of Sub-10-Nanometer Citrate-Stabilized Gold Nanoparticles and Related Optical Properties. *Chem. Mater.* **2016**, *28* (4), 1066–1075.

(2) Mansuriya, B. D.; Altintas, Z. Enzyme-Free Electrochemical Nano-Immunosensor Based on Graphene Quantum Dots and Gold Nanoparticles for Cardiac Biomarker Determination. *Nanomaterials* . 2021. https://doi.org/10.3390/nano11030578.

(3) Umpleby, R. J.; Baxter, S. C.; Chen, Y.; Shah, R. N.; Shimizu, K. D. Characterization of Molecularly Imprinted Polymers with the Langmuir− Freundlich Isotherm. *Anal. Chem.* **2001**, *73* (19), 4584–4591.

(4) Khoshfetrat, S. M.; Seyed Dorraji, P.; Shayan, M.; Khatami, F.; Omidfar, K. Smartphone-Based Electrochemiluminescence for Visual Simultaneous Detection of RASSF1A and SLC5A8 Tumor Suppressor Gene Methylation in Thyroid Cancer Patient Plasma. *Anal. Chem.* **2022**, *94* (22), 8005–8013.

(5) Love, J. C.; Estroff, L. A.; Kriebel, J. K.; Nuzzo, R. G.; Whitesides, G. M. Self-Assembled Monolayers of Thiolates on Metals as a Form of Nanotechnology. *Chem. Rev.* **2005**, *105* (4), 1103–1170. https://doi.org/10.1021/cr0300789.

(6) Drzazgowska, J.; Schmid, B.; Süssmuth, R. D.; Altintas, Z. Self-Assembled Monolayer Epitope Bridges for Molecular Imprinting and Cancer Biomarker Sensing. *Anal. Chem.* **2020**, *92* (7), 4798–4806.

(7) Barbero, C.; Silber, J. J.; Sereno, L. Formation of a Novel Electroactive Film by Electropolymerization of Ortho-Aminophenol: Study of Its Chemical Structure and Formation Mechanism. Electropolymerization of Analogous Compounds. *J. Electroanal. Chem. interfacial Electrochem.* **1989**, *263* (2), 333–352.
